# Day zero of autologous cryopreserved hematopoietic stem cell transplantation: microcosting of care provided by senior nurses*

**DOI:** 10.1590/1980-220X-REEUSP-2025-0528en

**Published:** 2026-03-16

**Authors:** Valéria Aparecida Gomes Nunes, Antônio Fernandes Costa Lima

**Affiliations:** 1 Universidade de São Paulo, Escola de Enfermagem, Programa de Pós-Graduação em Gerenciamento em Enfermagem, São Paulo, SP, Brazil.; 2 Universidade de São Paulo, Escola de Enfermagem, Departamento de Orientação Profissional, São Paulo, SP, Brazil.

**Keywords:** Hematopoietic Stem Cell Transplantation, Transplant Recipients, Hospital Care, Nursing Care, Direct Service Costs

## Abstract

**Objective:**

To measure the average direct cost of care provided by senior nurses to patients on day zero of autologous, cryopreserved hematopoietic stem cell transplantation in a public transplant hospital in the state of São Paulo.

**Method:**

Quantitative, exploratory-descriptive, single-case study based on bottom-up micro-costing, conducted from February to April 2025.

**Results:**

Twenty-eight procedures performed by senior nurses on 16 patients on day zero of the transplant were observed, covering the pre-infusion (n = 718), intra-infusion (n = 375), and post-infusion (n = 463) phases of hematopoietic stem cell transplantation. In the pre-infusion phase of hematopoietic stem cell transplantation, the total average direct cost was US$59.52; in the intra-infusion phase, US$663.45; and in the post-infusion phase, US$16.84. The average direct cost of nursing labor predominated in all three phases. However, in the intra-infusion phase, the cost of supplies was the largest contributor to the total average direct cost.

**Conclusion:**

Considering the three phases (n = 1556 observations), the total average direct cost was US$739.81 (100.00%), with 8.04% related to the pre-infusion phase, 89.68% to the intra-infusion phase, and 2.28% to the post-infusion phase.

## INTRODUCTION

Brazil ranks second worldwide in terms of the number of transplants. It is recognized as having the most comprehensive public organ and tissue transplant program in the world, mainly financed with public funds by the Unified Health System (SUS)^(1,2)^. According to the Brazilian Transplant Registry of the Brazilian Association of Organ and Tissue Transplants, in 2024, 26509 organ transplants and 3821 hematopoietic stem cell transplants (HSCT) were performed. Among the HSCTs, 2345 were autologous transplants and 1476 were allogeneic transplants. Of the total HSCTs, 1664 occurred in the city of São Paulo, of which 887 were autologous transplants and 777 were allogeneic transplants^(3)^.

HSCT is a highly complex, potentially curative elective therapeutic procedure capable of reconstituting the hematopoietic and immune systems in patients with malignant and non-malignant hematological, autoimmune, congenital, and metabolic diseases^(4)^. It is performed after bone marrow suppression through the administration of chemotherapy or radiotherapy doses, replacing unhealthy bone marrow (BM) cells with healthy hematopoietic stem cells (HSCs) to restore hematopoiesis. It requires specialized monitoring and management for both immediate and late complications that may occur in the long term.

BM was considered the main source for HSC collection; however, scientific advances have enabled the use of alternative sources, such as peripheral blood and umbilical cord and placental blood. HSCs can be obtained from two main sources: autologous, from BM and peripheral blood when collected from the patient themselves, or allogeneic sources, collected from BM, peripheral blood, and placental umbilical cord blood from a donor, who may or may not be related to the patient’s family^(4)^.

According to the Worldwide Network for Blood and Marrow Transplantation, the implementation of an HSCT service should be conducted in a structured, progressive manner, guided by the principles of quality and safety for the patient and donor. The requirements for establishing autologous and allogeneic HSCT programs may vary depending on the type of transplant, indications, availability of resources, and level of complexity involved. Among the essential elements for the viability of these programs are: adequate infrastructure, which should include facilities for apheresis, a cell processing laboratory or access to laboratory services for cell counting, cryopreservation, and storage of HSCs, as well as an isolation area for patients, and a multidisciplinary team specialized in hematology, chemotherapy management, hospital infection control, and intensive care, ensuring the safety and efficacy of the procedure^(5)^.

“Day zero” corresponds to the moment of HSC infusion and is the main stage of HSCT, requiring institutional protocols based on scientific evidence, which are crucial for reducing risks, improving care, promoting patient safety, and favoring the therapeutic success of HSCT^(6)^.

Nurses are responsible for infusing HSC and ensuring a series of essential care measures, including the preparation and administration of medications before, during, and after infusion; rigorous monitoring and constant surveillance to prevent, identify, and intervene in complications related to the procedure, generating costs that need to be identified and properly managed.

In healthcare organizations, nurses are key in the management of financial and care resources; due to their uninterrupted 24-hour work, they have detailed knowledge of patients’ needs and the resources necessary to meet the demands presented, which enables them to analyze and control the associated costs^(7)^.

An economic analysis study correlated data collected from 4513 HSCTs performed in 2017 in 13 of the 28 countries belonging to the Latin America Bone Marrow Transplant Group (Mexico, Cuba, Panama, Costa Rica, Colombia, Peru, Brazil, Ecuador, Venezuela, Uruguay, Paraguay, Chile, and Argentina). There was a variation from 12 HSCTs performed in Venezuela to 345 HSCTs in Uruguay; transplant rates were higher in countries where procedures cost more when performed in private for-profit hospitals and paid for with public funds. The costs of autologous HSCT ranged from US$65000 in Costa Rica to US$900000 in Panama; and the costs of related allogeneic HSCT ranged from US$150000 in Mexico to US$860000 in Uruguay. The study highlighted the complexity of accurately estimating the cost of HSCT in public hospitals, as it does not consider the labor costs of medical and nursing staff, nor the costs of many widely used medications, such as antibiotics, intravenous fluids, disposable supplies, among others, which are typically considered in private hospitals^(8)^.

Knowing the costs associated with the different phases of HSCT, notably in public health institutions in the context of the SUS, can assist in the formulation of treatment strategies, favoring the rational allocation of finite resources without compromising the desired quality, resulting in better outcomes for transplant patients.

From this perspective, the use of bottom-up micro-costing has been disseminated in the health sector because it supports the identification and direct valuation of each resource consumed, item by item (time spent by the professional, consumption of medications and materials), for each specific patient or service. Such information assists in the efficient allocation of resources, budget planning, and priority setting, contributing to the sustainability of the healthcare system. This method is useful for determining the costs of new technologies, treatments, or interventions; it provides robust, evidence-based data to inform the decision-making of managers, administrators, and healthcare policymakers^(9,10,11)^.

Considering that HSCT is a highly complex, high-cost procedure with a significant impact on health systems, especially in developing countries, there is significant inequality in access to this therapy. Despite the progressive growth in the number of HSCTs in Latin America in recent decades, the rates of performance remain seven to eight times lower than those observed in the United States and Europe, which vary by up to 30 times between countries in the region itself, due to socioeconomic, structural, and health system financing factors^(8)^. Furthermore, there are still few studies that objectively and in detail measure the direct costs associated with the critical stages of HSCT, particularly those related to nursing care, in which nurses play a central role in maintaining the safety and quality of care provided.

Thus, this study aims to measure the average direct cost (ADC) of care provided by senior nurses to patients on day zero of autologous cryopreserved hematopoietic stem cell transplantation (HSCT) at a public transplant hospital (PTH) in the state of São Paulo.

## METHOD

### Type or Study Design

This is a quantitative, exploratory/descriptive, single-case study.

### Location

The PTH, linked to the São Paulo State Health Secretariat and managed by a Social Health Organization, is a reference in different specialties, standing out in the areas of onco-hematology, hemophilia, urology, ophthalmology, nephrology, and neurosurgery. Its performance is based on the continuous improvement of care, patient quality and safety, and efficient management of organizational processes. It provides exclusive care through the SUS, ensuring regulated access based on technical criteria and distributive justice in the referral process for referred patients, carried out in an equitable, orderly, timely, and rational manner by the Health Services Supply and Regulation Center, with the availability and allocation of places operated through the São Paulo State Computerized Regulation System.

The PTH, with previous experience in structuring care pathways, consolidated from 2022 on a care pathway specifically focused on autologous or allogeneic HSCT, encompassing, in a continuous and integrated manner, all phases of multidisciplinary care, from pre-HSCT outpatient care and hospitalization for HSCT to post-HSCT outpatient care.

The Hematopoietic Stem Cell Transplant Unit (HSCTU) has 16 beds distributed across 14 rooms, 12 of which are single rooms with private bathrooms and two of which are double rooms with a shared bathroom. It has air conditioning, with temperature control between 18 and 21°C, and a protective air environment structure with positive pressure and a High Efficiency Particulate Arrestance filter to prevent fungal infections, and good practices for activities with HSC for therapeutic use in HSCT, fully complying with the requirements of RDC No. 508/2021 of the National Health Surveillance Agency^(12)^. Hospitalization is individualized and guided by patient-centered care and can include mobilization, collection, processing, conditioning, infusion of HSC, pancytopenia, leukocyte engraftment, and monitoring of complications until discharge, which comprise the therapeutic plan.

Due to the complexity and specificity of care at the HSCTU, only senior nurses work there (hired with at least 36 months of experience as a nurse and with specialization in the intended area of practice, under the Consolidation of Labor Laws), with a workload of 36 hours per week. Each of the work shifts (morning, afternoon, even night, and odd night) has seven senior nurses, totaling 28.

In 2024, 121 HSCTs were performed, of which 110 (90.9%) were autologous and 11 (9.1%) were allogeneic. The monthly distribution of these procedures ranged from 1 to 15 HSCTs, with a monthly average of 10.8 HSCTs. From January to September 2025, 81 HSCTs were performed, 72 (88.9%) of which were autologous and 9 (11.1%) allogeneic, with a monthly variation of 1 to 16 HSCTs and a monthly average of 9 HSCTs.

### Sample and Selection Criteria

An intentional non-probabilistic sampling was established with non-participant observation of at least 10.8 HSCTs (average monthly number of transplant patients in 2024), in the cryopreserved autologous modality, covering three typical months of care at the HSCTU. The procedures included were those provided by senior nurses to patients on day zero of autologous HSCT, from February to April 2025.

### Study Protocol

Prior to approval by the PTH Research Ethics Committee (CEP), consent was obtained from the Director and Nursing Manager of the institution. After CEP approval, the procedures performed by senior nurses on day zero, as well as the supplies (materials, medications, and solutions) required for their feasibility, were mapped from the electronic medical records of patients undergoing autologous cryopreserved HSCT, considering three typical months of operation.

One of the authors, who has been a Nursing Supervisor at PTH for seven years, conducted a review of the institutional routine, together with two nurses with extensive experience in BMT, aiming to ensure the standardization of the care provided by the senior nurse on day zero of HSCT.

The document entitled “Inpatient Unit Routine – BMT: Procedures on day zero of cryopreserved hematopoietic stem cell transplantation” was validated by senior nurses, analyzed, and approved by the Administrative, Medical, and Nursing Management and Boards of PTH. Subsequently, it was made available for consultation in the institutional Integrated Management System (SOGI in the Portuguese acronym) and the training of senior nurses working in the three shifts of the HSCTU was promoted, regarding the changes/adjustments required for its implementation.

After confirming that all senior nurses were adequately complying with the established routine, a data collection instrument was proposed, divided into: 1) Characterization of the recipient of cryopreserved autologous HSCT; 2) Stem cell infusion data – Day Zero; 3) Average Direct Cost (ADC) of procedures performed on day zero of HSCT with the infusion of cryopreserved autologous HSC; which was also reviewed, modified with the inclusion of suggestions, and validated by senior nurses. All senior nurses accepted the invitation to participate in the study. Upon signing the Free and Informed Consent Form, the author, Nursing Supervisor, proceeded to collect the data from February to April 2025, covering non-participating observations of autologous cryopreserved HSCT.

The study was based on direct costs, which refer to all those that can be identified and measured, related to a specific procedure, which can be attributed to a corresponding cost center, such as hospital supplies such as needles, syringes, catheters, medications, and direct labor (DL) costs of specific human resources^(13)^.

The bottom-up micro-costing method was used because it allows for a detailed definition of the cost variables, based on individual patient treatment data, with the review of medical records or specific clinical records for the study^(10,11)^.

DL costs refers to professionals who work directly on a given product or service, when it is possible to measure the time spent on its realization. It consists of salary, charges, vacation pay, and a 13th month salary^(13)^.

The average DL payroll for the 28 senior nurses was calculated based on average salaries (salaries, benefits, bonuses, and social charges) for four typical months of work at the HSCTU, provided by the Personnel Management Department. The values obtained were R$ 9162.02 for 144 hours, R$ 63.63 per hour, and R$ 1.06 per minute. The costs of materials and medications/solutions were provided by the Purchasing, Pharmacy, and Warehouse Departments of the PTH.

The ADC of the procedures performed by senior nurses on day zero of the cryopreserved autologous HSCT was obtained by multiplying the time spent (timed) by the unit cost of the DL and adding it to the cost of the inputs used^(14)^.

The equation $\bar{C(P_{t})}\;=\;\sum_{k=1}^{n}(\bar{q_{k}}.\bar{Pu_{k}})+\sum_{k=1}^{n}(\bar{qs_{k}.}\bar{PSu_{k}})+\sum_{c=1}^{n}(\bar{t_{c}}.{\bar{Su}}_{c});$ ; was used, defining the average quantity of materials is $\left[\bar{qm_{k}}\right]$ ; the average unit price of each material $\left[\bar{Pmu_{k}}\right]$ the average quantity of solutions/medications $\left[\bar{{\text{qS}}_{\text{k}}}\right]$ ; the average unit price of each solution/medication $\left[\bar{PSu_{k}}\right]$ ; the average time spent by each professional category $\left[\bar{t_{c}}\right]$ ; and the average unit wage bill of the DL for each professional category $\left[\bar{Su_{c}}\right]$ ^(14)^.

The Brazilian real (R$) was used, later converted to US dollars (US$) at an average rate of R$ 5.75/US$ 1.00, based on the exchange rates from February to April 2025, provided by the Central Bank of Brazil.

### Data Analysis and Processing

The data were systematized in spreadsheets and subsequently transferred to the bottom-up microcosting simulator^(15)^, developed by the Economic Dimension of Nursing Management Research Group. They were then processed using descriptive statistics of position (mean, minimum, maximum) and scale (standard deviation – SD).

### Ethical Aspects

The Ethics Committee (CEP) of the proposing institution approved the study through opinion No. 6.530.539, dated November 24, 2023.

## RESULTS

From February to April 2025, 28 senior nurses were observed while providing care to 16 (100.00%) patients undergoing autologous HSCT, in the cryopreserved modality, at the HSCTU. Male patients prevailed (56.25%), with multiple myeloma (75.00%), an average age of 58.63 (SD = 11.24) years, and self-declared white skin color (68.75%).

Among the 28 (100.00%) senior nurses, females predominated (92.86%), with a mean age of 36.46 (SD = 7.78) years, a mean training time of 7.93 (SD = 5.58) years, and an average experience in BMT of 4.86 (SD = 2.38) years. All senior nurses had at least 36 months of experience as nurses and specialization in oncology.

The first phase, pre-infusion of autologous cryopreserved HSCT, comprised the interval between 12:00 a.m. on day zero until the beginning of the afternoon, when the procedure of contacting the Cell Storage Center to confirm the start time of the HSCT infusion took place.

Table 1 shows that among the 11 procedures (100.00%) performed by senior nurses in the pre-infusion phase of HSCT, the highest total ADCs were: Disinfection and preparation of liquid heating equipment (US$ 25.55 – SD = 2.52), Preparation and administration of intravenous and subcutaneous medications (US$ 8.55 – SD = 3.57), Collection of laboratory tests (US$ 5.50 – SD = 5.42), and Assistance and performance of body hygiene and concurrent cleaning of the bed and furniture (US$ 5.28 – SD = 2.31). Considering the sum of the total ADCs for the 11 procedures, which resulted in 718 observations, the total ADC was US$59.52, corresponding to the pre-infusion phase of autologous HSCT in the cryopreserved modality. In most procedures (90.90%), the ADC with the DL of senior nurses prevailed in the composition of the total ADC.

**Table 1:** Distribution of costs in US dollars (USD) with DL, inputs (medications/solutions and materials) required in procedures performed by senior nurses in the pre-infusion phase of autologous, cryopreserved HSCT, according to the average direct cost (ADC), standard deviation (SD), minimum cost, maximum cost, 1st quartile, median, and 3rd quartile – São Paulo, SP, Brazil, 2025.

Procedures (number of observations)	ADC with DL from nurses US$*	ADC with supplies (medicines/solutions and materials) US$*	ADC total US$*	Standard deviation US$*	Minimum cost US$*	Maximum cost US$*	1st quartile US$*	Median US$*	3rd quartile US$*
1. Collection of laboratory tests (n = 19)	3.31	2.19	5.50	5.42	2.18	17.77	2.52	2.88	3.95
2. Measurement of vital signs, capillary blood glucose, and body weight (n = 120)	0.97	0.05	1.02	0.46	0.38	2.96	0.74	0.93	1.29
3. Intentional round (n = 57)	0.77	0.00	0.77	0.28	0.37	1.66	0.55	0.74	0.92
4. Assistance with and performance of personal hygiene (bathing) and cleaning of the bed and furniture (n = 48)	3.42	1.86	5.28	2.31	1.88	9.10	3.13	4.38	7.22
5. Assistance with bladder and bowel elimination, meals, and fluid intake (n = 85)	4.25	0.68	4.94	2.52	0.59	10.73	2.82	4.67	6.70
6. Testing the monitor, separating, organizing, and arranging emergency supplies and equipment (n = 55)	2.62	0.00	2.62	1.81	0.55	7.01	1.29	1.84	3.69
7. Disinfection and preparation of liquid heating equipment (n = 16)	10.90	14.64	25.55	2.52	20.47	28.58	23.92	25.96	27.59
8. Preparation and administration of IV and SC medications (n = 82)	4.64	3.92	8.55	3.57	3.18	26.81	5.68	6.97	8.30
9. Preparation and administration of oral medications (n = 145)	1.14	0.41	1.55	3.07	0.31	36.77	0.68	0.90	1.61
10. Central venous catheter dressing change, catheter flow and reflux test (n = 75)	2.49	0.62	3.09	1.15	1.31	5.82	2.04	2.76	3.28
11. Contact with the cell storage center (n = 16)	0.65	0.00	0.65	0.34	0.18	1.29	0.37	0.65	0.78

*Exchange rate: R$ 5.75/US$ 1.00. Central Bank, based on the average exchange rate from February to April 2025.

Regarding the consumption of medical supplies and solutions, regular insulin (US$24.89), NPH insulin (US$8.63), 1000ml distilled water (US$13.10), and anaerobic bottles (US$4.37) had the highest unit costs.

The second phase, intra-infusion of HSCT, comprised the infusion procedure, continuous monitoring, and strict control of vital signs, in addition to immediate intervention by the senior nurse, if necessary, due to the occurrence of adverse events. During the study, seven patients experienced adverse reactions, four of which were characterized by fever and systemic changes, resulting in the collection of laboratory tests, specifically blood culture, with a ADC of US$17.46. Among the supplies used, anaerobic bottles had a high unit cost ($52.38), corresponding to 89.68% of the ADC of the supplies consumed. Table 2 shows that of the 10 procedures (100.00%) performed, those with the highest total ADC were: HSC bag infusion (US$631.62 – SD = 1.66) and collection of laboratory tests (US$17.46 – SD = 0.24). Considering the sum of the total ADC of these 10 procedures, which resulted in 375 observations, the total ADC was US$663.45 (100.00%). Although the ADC with the DL of nurses predominated in most procedures (70.00%), the ADC with supplies was the largest contributor to the total ADC.

**Table 2:** Distribution of costs in US dollars (US$) with DL, supplies (medications/solutions and materials) required in procedures performed by senior nurses during the intra-infusion phase of HSC in Autologous, cryopreserved HSCT, according to average direct cost (ADC), standard deviation (SD), minimum cost, maximum cost, 1st quartile, median, and 3rd quartile and median – São Paulo, SP, Brazil, 2025.

Procedures (number of observations)	ADC with DL from nurses US$*	ADC with supplies (medicines/solutions and materials) US$*	ADC total US$*	Standard deviation US$*	Minimum cost US$*	Maximum cost US$*	1st quartile US$*	Median US$*	3rd quartile US$*
1. Measurement of vital signs, capillary blood glucose, and patient monitoring (n = 165)	0.49	0.03	0.52	0.27	0.19	1.86	0.38	0.38	0.56
2. Guidance and clarification on the HSC infusion procedure (n = 19)	1.28	0.00	1.28	0.54	0.55	2.58	0.92	1.29	1.47
3. Guidance and assistance with bladder and bowel control, and fluid intake (n = 14)	0.82	1.32	2.14	0.62	1.22	3.38	1.59	1.96	2.54
4. Concurrent cleaning of furniture (n = 16)	1.06	0.78	1.85	0.39	1.13	2.42	1.49	1.69	1.90
5. Double-checking and thawing of HSC bags (n = 90)	0.35	0.49	0.84	1.04	0.05	3.73	0.25	0.42	0.61
6. HSC bag infusion (n = 45)	3.27	628.36	631.62	1.66	628.10	637.13	630.68	631.23	632.23
7. Preparation and administration of medications (IV) (n = 4)	2.67	0.90	3.57	1.83	0.88	4.88	2.06	3.40	4.48
8. Collection of lab tests (n = 4)	2.86	14.60	17.46	0.24	16.68	17.24	16.90	17.04	17.14
9. Washing of equipment after HSC infusion (n = 16)	1.91	0.84	2.76	0.68	1.01	3.77	1.51	1.84	2.34
10. Central venous catheter dressing change, catheter flow and reflux test (n = 18)	0.97	0.45	1.41	0.35	0.37	2.11	1.21	1.36	1.41

*Exchange rate: R$ 5.75/US$ 1.00. Central Bank, based on the average exchange rate from February to April 2025.

Among the supplies consumed during the HSCT infusion phase, the HSC bag (US$627.91), anaerobic bottle (US$4.37), and comadre (US$1.10) had the highest unit costs.

The third phase, post-infusion of HSCT, covered the systematic observation of the procedures performed after washing the equipment, following the infusion of HSC, including all observations of the procedures performed at the end of the afternoon shift, extending until midnight on day zero.

Table 3 shows that among the seven procedures (100.00%) performed, the highest total ADCs were: Preparation and administration of intravenous and subcutaneous medications (US$6.77 – SD = 2.06), Concurrent cleaning of furniture (US$2.40 – SD = 0.35) and Assistance with bladder and bowel elimination, meals, and fluid control (US$2.29 – SD = 1.08). Considering the sum of the total ADCs for the seven procedures, which resulted in 463 observations, the total ADC was US$16.84. In most procedures (85.71%), the ADC with the DL of senior nurses prevailed in the composition of the total ADC.

**Table 3:** Distribution of costs in US dollars (USD) with DL, inputs (medications/solutions and materials) required in procedures performed by senior nurses in the post-infusion phase of autologous cryopreserved HSCT, according to average direct cost (ADC), standard deviation (SD), minimum cost, maximum cost, and median – São Paulo, SP, Brazil, 2025.

Procedures (number of observations)	ADC with DL from nurses US$*	ADC with supplies (medicines/solutions and materials) US$*	ADC total US$*	Standard deviation US$*	Minimum cost US$*	Maximum cost US$*	1st Quartile US$*	Median US$*	3rd Quartile US$*
1. Measurement of vital signs, capillary blood glucose, and body weight (n = 176)	0.77	0.03	0.80	0.26	0.38	2.27	0.58	0.74	0.93
2. Concurrent cleaning of furniture (n = 16)	1.11	1.30	2.40	0.35	1.65	2.81	1.99	2.20	2.55
3. Assistance with bladder and bowel elimination, meals, and fluid intake (n = 77)	1.64	0.65	2.29	1.08	0.79	5.64	1.34	2.46	2.90
4. Emptying and cleaning the liquid heating equipment (n = 16)	1.53	0.35	1.88	0.54	0.91	2.75	1.29	1.67	2.00
5. Preparation and administration of intravenous and subcutaneous medications (n = 55)	4.10	2.66	6.77	2.06	2.45	11.14	3.92	5.42	7.12
6. Preparation and administration of oral medications (n = 110)	0.81	0.17	0.98	0.73	0.28	3.07	0.49	0.67	1.01
7. Central venous catheter dressing change, catheter flow and reflux test (n = 13)	1.09	0.62	1.72	1.06	0.75	3.77	0.93	1.12	1.32

*Exchange rate: R$ 5.75/US$ 1.00. Central Bank, based on the average exchange rate from February to April 2025.This is an open-access article distributed under the terms of the Creative Commons Attribution License.

In the post-HSCT infusion phase, regular insulin (US$24.89), 1000 ml distilled water (US$13.10), and NPH insulin (US$8.63) were the supplies with the highest unit costs. The macro-drop equipment with side injector and air filter (US$2.81) was the most used material (42.19%) in the administration of hydration solutions.

## DISCUSSION

All procedures performed on “day zero” of the HSCT were performed exclusively by senior nurses with proven specialized clinical competence, demonstrating the PTH’s commitment to maintaining a highly qualified team, aiming at the safe performance of the essential stages of the HSCT. It is worth highlighting the indispensability of properly trained nurses at this critical moment of the transplant, since the success of the procedure, patient safety, and the prevention or minimization of adverse events depend directly on the technical expertise of these professionals.

Nurses play a central role in the care of patients undergoing HSCT, and their technical expertise is crucial for safety, prevention of complications, and qualified conduct of the required procedures^(16,17)^.

In the pre-infusion phase, 59.07% of the total ADC corresponded to DL for senior nurses; the most significant costs (US$25.55) were due to the disinfection and preparation of the liquid heating equipment. It should be clarified that, with regard to cryopreserved HSC infusion practices, the thawing of bags requires hospital equipment and carefully validated techniques (strict temperature control, adequate cleaning and disinfection, and sterile technique) to ensure cell viability, minimize the risk of contamination, and ensure the safety of the recipient^(18)^.

The administration of intravenous and subcutaneous medications (ADC = US$ 8.55) stood out in the composition of the total ADC in the pre-infusion phase, being a necessary procedure for clinical management, through pharmacological measures aimed at preventing adverse events (AE) related to the infusion.

A study that analyzed 1.269 cryopreserved autologous HSC infusions, containing bags with the cryoprotective reagent dimethyl sulfoxide (DMSO), in 1.191 adult patients found that 37.8% of infusions had some AE. DMSO was associated with most reactions, reinforcing those clinical interventions, especially adjustments and administration of pre-infusion medications, can reduce toxicity and increase transplant safety. The frequency of these AEs, classified as mild or moderate, indicates the relevance of preventive strategies, such as adequate hydration, use of antihistamines, and antiemetics, capable of mitigating risks and improving the safety of HSC infusion^(19)^. However, among the medications used in this study, regular insulin (US$ 24.89) had the highest ADC, due to pre-existing comorbidities that required glycemic adjustments for the safe clinical management of transplant patients.

The ADCs for collecting laboratory tests ($5.50); assistance with and performance of personal hygiene and concurrent cleaning of the bed and furniture ($5.28) also had an impact on the total ADC for the pre-infusion phase. A systematic review^(20)^ covering studies conducted in hematopoietic cell transplant units showed that several pathogens were directly associated with environmental sources, viral outbreaks, and inadequate disinfection practices. These findings indicate that good hygiene, cleaning, and disinfection practices are an essential investment to prevent infections that can result in clinical worsening, prolonged hospital stays, and significantly increased healthcare costs^(20)^.

Among the procedures performed during the intra-infusion phase, the infusion of the HSC bag (95.20%) contributed most to the total ADC (US$663.45 – 100.00%), followed by the collection of laboratory tests (2.63%).

A study^(21)^ that analyzed the complete cycle of collection, cryopreservation, storage, and use of HSC in a European transplant center over 12 years showed that the collection, processing, storage, and disposal of HSC involves substantial and often underestimated costs, especially when considering bags that are never used in transplants. It found that HSC infusion occurs mostly within a short time after collection and that the management of bags stored for long periods results in high costs for the services^(21)^.

Another study^(22)^, which evaluated 613 patients with multiple myeloma for nine years, found that the utilization rate of bags stored for more than 30 days was extremely low (14.9%), and that 69.00% of patients kept cryopreserved HSC bags in stock for two years or more, with significant costs associated with maintenance in liquid nitrogen, additional apheresis procedures, and late disposal. Most of the costs related to the HSC cycle are not concentrated at the time of infusion, but in the previous stages of mobilization, collection, and prolonged storage, which are standardized processes that directly influence the final value of the inputs used at the time of bedside administration^(22)^.

Both of the above-mentioned studies^(21,22)^ highlight that healthcare institutions need to review their practices and workflows related to HSC management to optimize resources, reduce waste, and ensure the economic and operational sustainability of HSCT.

During the infusion phase of HSC, the occurrence of adverse reactions required the collection of laboratory tests, and anaerobic blood culture bottles were high-cost items. A narrative review study highlighted that most blood cultures collected in a hospital setting, with indications based on fever or nonspecific changes, have a low correlation with bacterial contamination and emphasized that indiscriminate collection contributes to increased costs^(23)^. Thus, although investigating fever during the infusion of HSC is clinically relevant, careful indication of blood culture is essential to balance diagnostic accuracy, patient safety, and rational use of supplies, particularly those with high unit costs.

In the post-infusion phase of HSC, the preparation and administration of medications (US$ 6.77 – 40.20%) corresponded to the highest ADC. The most commonly used material in the administration of hydration solutions was the macro-drop equipment with a side injector and air filter (US$2.81) together with 0.9% saline solution 1000 mL (US$1.07). This is because a good practice adopted at the HSCTU consists of replacing the equipment each time a hydration solution is prepared, associated with the use of a continuous infusion pump. The emphasis on hydration after infusion of cryopreserved HSC in DMSO is justified, as a narrative review study described that infusion of HSC with DMSO is related to nephrotoxic, cardiovascular, neurological, and gastrointestinal effects^(24)^.

A study^(25)^ investigated the variation in intravenous hydration practices after infusion of autologous HSCs cryopreserved in DMSO in 510 transplant centers affiliated with the European Bone Marrow Transplantation. The results showed that post- infusion hydration is widely used to protect renal function and aid in the elimination of DMSO from the body; however, there is still no scientific evidence to confirm its effectiveness in reducing associated toxicity^(25)^.

The second procedure with the highest ADC in the post- infusion phase was the concurrent cleaning of furniture (US$ 2.40). This value is lower than that obtained in the pre-infusion phase of the HSC (US$ 5.28), since, in this phase, the cost does not involve referral and assistance for the patient’s personal hygiene.

Although the ADC with supplies was the largest contributor to the total ADC composition of the intra-HSCT phase, the ADC with the DL of senior nurses prevailed in all three phases. Studies^(17,18,19,20,21,22,23,24,25,26)^ reinforce that nurses are the professionals who work most closely with patients during all phases of HSCT, which is highly complex and permeated by intense emotional stress and high clinical risk, performing roles that include clinical surveillance, management of adverse effects, emotional support, and therapeutic education that require high professional expertise^(17,18,19,20,21,22,23,24,25,26)^. These activities, which were also observed in this study, justify why the DL of nurses represented the largest portion of the ADC.

It is evident that nursing care in HSCT has a direct impact on the prevention of complications, especially through systematic clinical surveillance, the implementation of safety protocols, and early monitoring of toxicological and infectious effects. This demonstrates that the standardization of nursing procedures in autologous stem cell infusion reduces variability in care and contributes to safer clinical outcomes^(6)^. Continuous presence, systematic surveillance, the provision of essential care, and the prevention of AE place nurses at the operational and care center of the care required in HSCT^(6,16,26,27)^. It should be emphasized that the costs associated with the work of nurses should not be interpreted as a marker of expense, but rather as an essential investment in patient safety and quality of care. Thus, the findings of this study corroborate the evidence that the time spent by nurses in direct care is an indispensable component for the safety of HSCT.

### Implications for Practice

Knowing the costs related to nursing care in the different phases/stages of day zero of HSCT, notably in public health institutions in the context of the SUS, can assist in the formulation of strategies that also cover financial aspects, prioritizing the review of processes and the rational allocation of finite resources, without compromising the desired quality, resulting in better outcomes for patients.

### Study Limitations

A limitation is the non-participant observation restricted to procedures related to autologous HSCT in the cryopreserved modality, which does not allow the application of the results obtained to other types of HSCT or infusion modalities.

## CONCLUSION

The total ADC of the care provided by senior nurses to patients on day zero of HSCT, based on 1.556 observations of 28 procedures, corresponded to US$739.81 (100.00%), with 8.04% related to the pre-HSCT phase, 89.68% to the intra- HSCT phase, and 2.28% to the post-HSCT phase. In all three phases, ADC with DL by nurses predominated in the composition of total ADC. However, in the intra-HSCT phase, the ADC with supplies was the largest contributor to the total ADC composition, with the HSC bag standing out (unit value of US$627.91).

## DATA AVAILABILITY

Not applicable. This is a single case study whose dataset supporting the results is not publicly available due to the presence of sensitive information and the need to preserve the confidentiality of the participants. All information relevant to understanding the findings is described in the body of the article.

## References

[1] Brasil. Ministério da Saúde (2022). Saúde e Vigilância Sanitária. Doação de Órgãos. Brasil é o segundo maior transplantador de órgãos do mundo
[Internet].

[2] Brasil. Ministério da Saúde (2025). Atenção Especializada à Saúde. Sistema Nacional de Transplantes [Internet].

[3] Associação Brasileira de Transplantes de Órgãos (2025). Registro Brasileiro de Transplantes [Internet]. https://site.abto.org.br/wp-content/uploads/2025/05/rbt-n4-2024-populacao.pdf.

[4] Bazinet A, Popradi G (2019). A general practitioner’s guide to hematopoietic stem-cell
transplantation. Curr Oncol.

[5] Pasquini MC, Srivastava A, Ahmed SO, Aljurf M, Atsuta Y, Doleysh C (2020). Worldwide Network for Blood and Marrow Transplantation (WBMT)
recommendations for establishing a hematopoietic cell transplantation
program (Part I): minimum requirements and beyond. Hematol Oncol Stem Cell Ther.

[6] Figueiredo TWB, Mercês NNA, Silva LAA, Machado CAM (2019). Protocolo de cuidados de enfermagem no dia zero do transplante de
células-tronco hematopoéticas: construção coletiva. Texto Contexto Enferm.

[7] Lima AFC, Castilho V, Fugulin FMT, Kurcgant P (2023). Gerenciamento em enfermagem.

[8] Jaimovich G, Gale RP, Hanesman I, Vazquez A, Hammerschlak N, Simoes BP (2021). The paradox of haematopoietic cell transplant in Latin
America. Bone Marrow Transplant.

[9] Xu X, Lazar CM, Ruger JP (2021). Micro-costing in health and medicine: a critical
appraisal. Health Econ Rev.

[10] Etges APBS, Schlatter RP, Neyeloff JL, Araujo DV, Bahia LR, Cruz L (2019). Estudos de Microcusteio aplicados a avaliações econômicas em
saúde: uma proposta metodológica para o Brasil. J Bras Econ Saude.

[11] Brasil. Ministério da Saúde (2021). Diretriz Metodológica: estudos de microcusteio aplicados a avaliações
econômicas em saúde.

[12] Brasil. Ministério da Saúde (2021). Agência Nacional de Vigilância Sanitária. Resolução da Diretoria
Colegiada – RDC nº 508, de 27 de maio de 2021. Dispõe sobre as boas práticas em células humanas para uso terapêutico e
pesquisa clínica [Internet].

[13] Martins E (2018). Contabilidade de custos.

[14] Lima AFC (2017). Direct cost of monitoring conventional hemodialysis conducted by
nursing professionals. Rev Bras Enferm.

[15] Berger S, Saba A, Lima AFC (2025). Desenvolvimento do protótipo de um simulador de microcusteio
bottom-up para aplicação em Saúde e Enfermagem. Rev. Contemp.

[16] Fauer AJ, Choi SW, Friese CR (2019). The roles of nurses in Hematopoietic Cell Transplantation for
leukemia in older adults. Semin Oncol Nurs.

[17] Izu M, Silvino ZR, Santos LM, Balbino CM (2021). Nursing care for patients undergoing hematopoietic stem cell
transplantation. Acta Paul Enferm.

[18] Berz D, McCormack EM, Winer ES, Colvin GA, Quesenberry PJ (2007). Cryopreservation of hematopoietic stem cells. Am J Hematol.

[19] Otrock ZK, Sempek DS, Carey S, Grossman BJ (2017). Adverse events of cryopreserved hematopoietic stem cell
infusions. Transfusion.

[20] Kakoullis L, Chedid G, Walker B, Xirou V, Hafez S, Zisis SN (2025). Outbreaks in hematopoietic stem cell transplant units: a
systematic review. Infect Control Hosp Epidemiol.

[21] Kriegsmann K, Wack M, Pavel P, Schmitt A, Kriegsmann M, Bruckner T (2019). Collection, cryostorage, transplantation, and disposal of
hematopoietic stem cell products. Biol Blood Marrow Transplant.

[22] Benjamin CL, Desai S, Pereira D, Beitinjaneh A, Jimenez A, Goodman M (2023). Cryopreservation and storage patterns of hematopoietic progenitor
stem cells for multiple myeloma. Transfus Apher Sci.

[23] Fabre V, Carroll KC, Cosgrove SE (2022). Blood culture utilization in the hospital setting: a call for
diagnostic stewardship. J Clin Microbiol.

[24] Shu Z, Heimfeld S, Gao D (2014). Hematopoietic SCT with cryopreserved grafts: adverse reactions
after transplantation and cryoprotectant removal before
infusion. Bone Marrow Transplant.

[25] Kortleve J, Kisch A, Piepenbroek B, Mooyaart J, Kozijn A, Sohne M (2023). Variation in hydration use after reinfusion of autologous stem
cells in dimethyl sulfoxide (DMSO): a survey of EBMTcenters on behalf of the
EBMT Nurses Group. Res Square.

[26] Sayadi L, Zamanzadeh V, Valizadeh L, Taleghani F (2019). Caring process in hematopoietic stem cell transplantation: a
grounded theory study. Int J Hematol Oncol Stem Cell Res.

[27] Benicá TOS, Nascimento SCT, Pereira GC, Ramos LGA (2021). The role of nurses in hematopoietic stem cell
transplantation. Res Soc Dev.

