# Supplementary material for: Day zero of autologous cryopreserved hematopoietic stem cell transplantation: microcosting of care provided by senior nurses
Source: Rev Esc Enferm USP. 2026 Mar 16;60:e20250528. doi: 10.1590/1980-220X-REEUSP-2025-0528en (PMC12991409; doi:10.1590/1980-220X-REEUSP-2025-0528en)
Supplement: Translation

# Translation: Dia zero do transplante autólogo de células-tronco hematopoiéticas criopreservadas: microcusteio da assistência prestada por enfermeiras seniores*

**DOI:** 10.1590/1980-220X-REEUSP-2025-0528pt

**Authors:** Valéria Aparecida Gomes Nunes, Antônio Fernandes Costa Lima

**Affiliations:** 1 Universidade de São Paulo, Escola de Enfermagem, Programa de Pós-Graduação em Gerenciamento em Enfermagem, São Paulo, SP, Brasil.; 2 Universidade de São Paulo, Escola de Enfermagem, Departamento de Orientação Profissional, São Paulo, SP, Brasil.

**Keywords:** Transplante de Células-Tronco Hematopoiéticas, Transplantados, Assistência Hospitalar, Cuidados de Enfermagem, Custos Diretos de Serviços

## Abstract

**Objetivo:**

Mensurar o custo direto médio da assistência prestada por enfermeiros seniores à pacientes no dia zero do transplante de células-tronco hematopoiéticas autólogo, criopreservado, em um hospital público de transplantes do Estado de São Paulo.

**Método:**

Pesquisa quantitativa, exploratório-descritiva, do tipo estudo de caso único, fundamentada no microcusteio *bottom-up*, conduzida de fevereiro a abril/2025.

**Resultados:**

Foram observados 28 procedimentos realizados por enfermeiros seniores à 16 pacientes no dia zero do transplante abrangendo as fases pré (n = 718), intra (n = 375) e pós infusão (n = 463) de células-tronco hematopoiéticas. Na fase pré infusão do transplante de células-tronco hematopoiéticas obteve-se o custo direto médio total de US$59,52; na fase intra infusão US$663,45 e na fase pós infusão US$16,84. Houve o predomínio do custo direto médio com a mão de obra dos enfermeiros nas três fases. Contudo, na fase intra infusão o custo com insumos foi o de maior contribuição na composição do custo direto médio total.

**Conclusão:**

Considerando as três fases (n = 1556 observações), o custo direto médio total correspondeu a US$739,81 (100,00%) sendo 8,04% relativos à fase pré infusão, 89,68% à fase intra e 2,28% à fase pós infusão.

## INTRODUÇÃO

O Brasil se consolida mundialmente como o segundo país em número de transplantes. É reconhecido como detentor do programa público de transplantes de órgãos e tecidos mais abrangente do mundo, majoritariamente financiado com recursos públicos pelo Sistema Único de Saúde (SUS)^(1,2)^. De acordo com o Registro Brasileiro de Transplantes da Associação Brasileira de Transplantes de Órgãos e Tecidos, em 2024, foram realizados 26.509 transplantes de órgãos e 3.821 Transplantes de Células-Tronco Hematopoiéticas (TCTH). Dentre os TCTH, 2.345 foram transplantes autólogos e 1.476 transplantes alogênicos. Do total de TCTH, 1.664 ocorreram na cidade de São Paulo dos quais 887 foram transplantes autólogos e 777 transplantes alogênicos^(3)^.

O TCTH consiste em um procedimento terapêutico eletivo de alta complexidade, potencialmente curativo, capaz de reconstituir o sistema hematopoiético e imunológico em pacientes com doenças hematológicas malignas e não malignas, autoimunes, congênitas e metabólicas^(4)^. É realizado após a supressão medular, por meio da administração de doses de quimioterapia ou radioterapia, com a substituição das células insalubres da medula óssea (MO) por Células-Tronco Hematopoiéticas (CTH) saudáveis para o restabelecimento da hematopoiese. Requer vigilância e manejo especializado tanto para as complicações imediatas quanto para as complicações tardias que podem ocorrer a longo prazo.

A MO era considerada a principal fonte de coleta para obtenção das CTH, contudo, os avanços científicos possibilitaram a utilização de fontes alternativas, como o sangue periférico e sangue do cordão umbilical e placentário. As CTH podem ser obtidas a partir de duas fontes principais: autólogas, provenientes da MO e do sangue periférico quando coletadas do próprio paciente, ou fontes alogênicas, coletadas da MO, sangue periférico e sangue do cordão umbilical placentário de um doador, que pode ser relacionado ou não à família do paciente^(4)^.

Segundo o *Worldwide Network flor Blood and Marrow Transplantation*, a implementação de um serviço de TCTH deve ser conduzida de forma estruturada, progressiva e orientada pelos princípios de qualidade e segurança do paciente e do doador. Os requisitos para o estabelecimento de programas de TCTH autólogo e alogênico podem variar dependendo do tipo de transplante, das indicações, da disponibilidade de recursos e do nível de complexidade envolvido. Entre os elementos imprescindíveis para a viabilidade desses programas destacam-se: infraestrutura adequada, a qual deve incluir instalações para a realização de aférese, laboratório de processamento celular ou acesso a serviços laboratoriais para contagem de células, criopreservação e armazenamento das CTH, bem como área de isolamento para pacientes, e uma equipe multiprofissional especializada em hematologia, manejo de quimioterapia, controle de infecção hospitalar e cuidados intensivos assegurando a segurança e eficácia do procedimento^(5)^.

O “dia zero” corresponde ao momento da infusão das CTH e corresponde à etapa principal do TCTH, requer protocolos institucionais fundamentados em evidências científicas, sendo determinante para reduzir riscos, qualificar a assistência, promover a segurança do paciente e favorecer o êxito terapêutico do TCTH^(6)^.

O enfermeiro é o responsável por infundir as CTH e assegurar uma série de cuidados essenciais, incluindo o preparo e a administração de medicações antes, durante e após a infusão; rigorosa monitorização e vigilância constante a fim de prevenir, identificar e intervir nas complicações relacionadas ao procedimento^(6)^, gerando custos que precisam ser identificados e adequadamente gerenciados.

Nas organizações de saúde, os enfermeiros são fundamentais no gerenciamento dos recursos financeiros e assistenciais; devido à sua atuação ininterrupta nas 24 horas, possuem um conhecimento detalhado das necessidades dos pacientes e dos recursos necessários para atender às demandas apresentadas, o que os capacita para atuar na análise e no controle dos custos associados^(7)^.

Estudo de análise econômica correlacionou o levantamento de dados de 4.513 TCTH, realizados em 2017, em 13 dos 28 países pertencentes ao *Latin America Bone Marrow Transplant Group* (México, Cuba, Panamá, Costa Rica, Colômbia, Peru, Brasil, Equador, Venezuela, Uruguai, Paraguai, Chile e Argentina). Verificou-se a variação de 12 TCTH realizados na Venezuela a 345 TCTH no Uruguai; as taxas de transplantes foram mais altas nos países onde os procedimentos custavam mais, quando eram feitos em hospitais privados com fins lucrativos e pagos com fundos públicos. Os custos de TCTH Autólogo variaram de US$ 65,000 na Costa Rica a US$ 900,000 no Panamá; e os custos de um TCTH Alogênico aparentado variaram de US$ 150,000 no México a US$ 860,00 no Uruguai. Destacou a complexidade de estimar com precisão o custo do TCTH em hospitais públicos, por não ser considerado o custo de mão de obra da equipe médica e de enfermagem, nem os custos de muitos medicamentos amplamente utilizados, como antibióticos, fluidos intravenosos, insumos descartáveis, entre outros; os quais são tipicamente considerados em hospitais privados^(8)^.

Conhecer os custos associados às diferentes fases do TCTH, notadamente em instituições públicas de saúde no contexto do SUS, pode auxiliar na formulação de estratégias de tratamento, privilegiando a alocação racional de recursos finitos, sem comprometimento da qualidade desejada, resultando em melhores desfechos para os pacientes transplantados.

Nesta perspectiva, o uso do microcusteio *bottom-up* tem sido disseminado na área da Saúde por subsidiar a identificação e valoração direta de cada recurso consumido, item por item (tempo despendido pelo profissional, consumo de medicamentos e materiais), para cada paciente ou serviço específico. Tais informações auxiliam na alocação eficiente de recursos, no planejamento orçamentário e na definição de prioridades, contribuindo para a sustentabilidade do sistema de saúde. Este método é útil para apurar os custos de novas tecnologias, tratamentos ou intervenções; fornece dados robustos e baseados em evidências para fundamentar a tomada de decisões de gerentes, gestores e formuladores de políticas de saúde^(9,10,11)^.

Considerando que o TCTH é um procedimento de alta complexidade, elevado custo e impacto significativo sobres os sistemas de saúde, especialmente em países em desenvolvimento, constata-se importante desigualdade no acesso a essa terapia. Apesar do crescimento progressivo do número de TCTH nas últimas décadas na América Latina, as taxas de realização permanecem de sete a oito vezes inferiores em comparação com as taxas observadas nos Estados Unidos e na Europa, que apresentam amplitude de variação de até 30 vezes entre países da própria região, em função de fatores socioeconômicos, estruturais e de financiamento do sistema de saúde^(8)^. Ademais, ainda são escassos os estudos que mensuram, de forma objetiva e detalhada, os custos diretos associados às etapas críticas do TCTH, particularmente aqueles relacionados à assistência de enfermagem, cujos enfermeiros desempenham papel central na manutenção da segurança e na qualidade do cuidado prestado.

Assim, este estudo objetiva mensurar o custo direto médio (CDM) da assistência prestada por enfermeiros seniores à pacientes no dia zero do transplante de células-tronco hematopoiéticas (TCTH) autólogo, criopreservado, em um hospital público de transplantes (HPT) do Estado de São Paulo.

## MÉTODO

### Tipo ou Desenho do Estudo

Trata-se de pesquisa quantitativa, exploratório/descritiva, do tipo estudo de caso único.

### Local

O HPT, vinculado à Secretaria de Saúde do Estado de São Paulo e gerenciado por uma Organização Social de Saúde, é referência em diferentes especialidades, destacando-se nas áreas de onco-hematologia, hemofilia, urologia, oftalmologia, nefrologia e neurocirurgia. Sua atuação está alicerçada na melhoria contínua da assistência, na qualidade e segurança do paciente e na gestão eficiente dos processos organizacionais. Presta atendimento exclusivo pelo SUS assegurando o acesso regulado com base em critérios técnicos e de justiça distributiva no processo de encaminhamento dos pacientes referenciados, realizado de forma equânime, ordenada, oportuna e racional pela Central de Oferta e Regulação de Serviços de Saúde, com disponibilização e alocação das vagas operacionalizadas por intermédio do Sistema Informatizado de Regulação do Estado de São Paulo.

O HPT, com experiência prévia na estruturação de linhas de cuidado assistenciais, consolidou, a partir de 2022, uma linha de cuidado voltada especificamente para o TCTH, autólogo ou alogênico, englobando, de forma contínua e integrada, todas as fases de atendimento multiprofissional, desde a ambulatorial pré-TCTH, internação hospitalar para a realização do TCTH, até a de atendimento ambulatorial pós-TCTH.

A Unidade de internação do transplante de células-tronco hematopoiéticas (UITCTH) dispõe de16 leitos de internação distribuídos em 14 quartos, sendo 12 de leito único e banheiro privativo e dois com dois leitos e um banheiro coletivo. Possui ambiente climatizado, com controle de temperatura entre 18 e 21°C, e estrutura de ar ambiente protetor com pressão positiva e filtro *High Efficiency Particulate Arrestance*, para prevenção de infecções fúngicas, e boas práticas para atividades com CTH para uso terapêutico no TCTH atendendo, integralmente, as exigências da RDC nº 508/2021 da Agência Nacional de Vigilância Sanitária^(12)^. A internação é individualizada e norteada pelo cuidado centrado no paciente e pode contemplar desde a mobilização, coleta, processamento, condicionamento, infusão de CTH, pancitopenia, enxertia leucocitária, e monitoramento de complicações até a alta, as quais compreendem o plano terapêutico.

Devido à complexidade e especificidade assistencial da UITCTH, nela atuam somente enfermeiros seniores (contratados com, no mínimo, 36 meses de experiência como enfermeiro e com especialização na área de atuação pretendida, em regime Consolidação das Leis do Trabalho), com carga de trabalho de 36 horas semanais. Cada um dos turnos de trabalho (manhã, tarde, noite par e noite ímpar) dispõe de sete enfermeiros seniores, totalizando 28.

Em 2024, foram realizados 121 TCTHs, dos quais 110 (90,9%) corresponderam à modalidade autóloga e 11 (9,1%) à modalidade alogênica. A distribuição mensal desses procedimentos variou de 1 a 15 TCTHs, apresentando uma média de 10,8 TCTHs mensais. De janeiro a setembro de 2025, foram realizados 81 TCTHs, sendo 72 (88,9%) autólogos e 9 (11,1%) alogênicos; com variação mensal de 1 a 16 TCTHs e média de 9 TCTHs mensais.

### Amostra e Critérios de Seleção

Estabeleceu-se uma amostragem não probabilística intencional com a observação não participante de, no mínimo, 10,8 TCTHs (quantitativo médio mensal de pacientes transplantados em 2024), na modalidade autóloga criopreservada, abrangendo três meses típicos de atendimento da UITCTH. Foram incluídos os procedimentos integrantes da assistência prestada por enfermeiros seniores à pacientes no dia zero do TCTH autólogo, no período de fevereiro a abril de 2025.

### Protocolo do Estudo

Previamente a aprovação do Comitê de Ética em Pesquisa (CEP) do HPT, obteve-se a anuência do Diretor e do Gerente de Enfermagem da Instituição. Após aprovação do CEP, foram mapeados, a partir dos prontuários eletrônicos dos pacientes submetidos ao TCTH na modalidade autólogo criopreservado, considerando três meses típicos de funcionamento, os procedimentos realizados por enfermeiros seniores no dia zero, bem como os insumos (materiais, medicamentos e soluções) requeridos à sua viabilização.

Uma das autoras, que é Supervisora de Enfermagem, há sete anos, no HPT, conduziu uma revisão da rotina institucional, juntamente com duas enfermeiras com ampla experiência em TMO, visando assegurar a padronização da assistência realizada pelo enfermeiro sênior no dia zero do TCTH.

O documento denominado “Rotina da Unidade de Internação – TMO: Procedimentos no dia zero do Transplante de Células-Tronco Hematopoiéticas na modalidade criopreservado”, foi validado pelos enfermeiros seniores, analisado e aprovado pelas Gerências e Diretorias Administrativa, Médica e de Enfermagem do HPT. Posteriormente, foi disponibilizado para consulta no Sistema de Gestão Integrado (SOGI) institucional e favorecida a capacitação dos enfermeiros seniores atuantes nos três turnos da UITCTH, quanto às mudanças/ajustes requeridos à sua implementação.

Após a constatação de que todos os enfermeiros seniores estavam cumprindo, adequadamente, a rotina estabelecida, propôs-se um instrumento para a coleta de dados dividido em: 1) Caracterização do(a) receptor(a) do TCTH criopreservadas na modalidade autóloga; 2) Dados da infusão das células-tronco – Dia Zero; 3) CDM dos procedimentos realizados no dia zero do TCTH com a infusão das CTH criopreservadas na modalidade autóloga; que também foi apreciado, modificado com a inclusão de sugestões e validado pelos enfermeiros seniores. Todos os enfermeiros seniores aceitaram o convite para participar do estudo. Mediante a assinatura do Termo de Consentimento Livre e Esclarecido, a autora Supervisora de Enfermagem procedeu a coleta dos dados, de fevereiro a abril de 2025, abrangendo as observações não participantes dos TCTH criopreservadas na modalidade autóloga.

O estudo fundamentou-se nos custos diretos, que dizem respeito a todos aqueles passíveis de identificação e mensuração, relativos a um determinado procedimento, que podem ser atribuídos a um centro de custo correspondente, à exemplo os insumos hospitalares tais como: agulhas, seringas, sondas, medicamentos e mão de obra direta (MOD) de recursos humanos específicos^(13)^.

Utilizou-se o método de microcusteio *bottom-up* por possibilitar definir, detalhadamente, as variáveis componentes dos custos, a parir de dados individuais do tratamento do paciente, com a revisão de prontuários ou de ficha clínica específica para o estudo^(10,11)^.

A MOD refere-se aos profissionais que trabalham diretamente sobre um determinado produto ou serviço, quando possível mensurar o tempo despendido à sua realização. É composta pelo salário, encargos, férias e 13º salário^(13)^.

Calculou-se a média da massa salarial da MOD dos 28 enfermeiros seniores a partir dos salários médios (salários, benefícios, gratificações e encargos sociais) relativos a quatro meses típicos de trabalho na UITCTH, disponibilizados pelo Departamento de Gestão de Pessoas. Então, obtiveram-se os valores de R$ 9.162,02 por 144 horas, R$ 63,63 por hora e R$ 1,06 por minuto. Os custos dos materiais e medicamentos/soluções foram fornecidos pelos Setores de Compras, Farmácia e Almoxarifado do HPT.

O CDM dos procedimentos realizados por enfermeiros seniores no dia zero do TCTH autólogo criopreservado foi obtido multiplicando-se o tempo despendido (cronometrado) pelo custo unitário da MOD e somando-o ao custo dos insumos utilizados^(14)^.

Utilizou-se a equação $\bar{C\left(P_{t}\right)}=\sum_{k=1}^{n}\left({\bar{q}}_{k}\cdot \bar{Pu_{k}}\right)+\sum_{k=1}^{n}\left({\bar{qS}}_{k}\cdot \bar{PSu_{k}}\right)+\sum_{c=1}^{n}({\bar{t}}_{c}\cdot \bar{Su_{c}}$ ; definindo-se a quantidade média dos materiais $\left[\bar{qm_{k}}\right]$ ; o preço unitário médio de cada material $\left[\bar{Pmu_{k}}\right]$ ; a quantidade média das soluções/medicamentos $\left[\bar{qS_{k}}\right]$ ; o preço unitário médio de cada solução/medicamento $\left[\bar{PSu_{k}}\right]$ ; o tempo médio de dedicação de cada categoria profissional $\left[\bar{t_{c}}\right]$ e a massa salarial unitária média da MOD de cada categoria profissional $\left[\bar{Su_{c}}\right]$ ^(14)^.

Empregou-se a moeda brasileira real (R$), posteriormente convertida para o dólar americano (US$) pela taxa média de R$ 5,75/US$ 1.00, com base nas cotações de fevereiro a abril de 2025, fornecidas pelo Banco Central do Brasil.

### Análise e Tratamento dos Dados

Os dados foram sistematizados em planilhas eletrônicas e posteriormente transferidos para o Simulador de microcusteio bottom-up^(15)^, desenvolvido pelo Grupo de Pesquisa Dimensão Econômica de Gerenciamento em Enfermagem. Em seguida, foram tratados por meio de estatística descritiva de posição (média, mínimo, máximo) e de escala (desvio padrão – DP).

### Aspectos Éticos

O CEP da Instituição proponente aprovou a realização do estudo por meio do parecer consubstanciado nº: 6.530.539, de 24/11/2023.

## RESULTADOS

De fevereiro a abril de 2025, foram observados 28 enfermeiros seniores durante a assistência prestada a 16 (100,00%) pacientes submetidos ao TCTH autólogo, na modalidade criopreservada, na UITCTH. Prevaleceram pacientes do sexo masculino (56,25%), com Mieloma Múltiplo (75,00%), média de idade de 58,63 (DP = 11,24) anos e autodeclarados de cor da pele branca (68,75%).

Dentre os 28 (100,00%) enfermeiros seniores, predominou o sexo feminino (92,86%), com média de idade de 36,46 (DP = 7,78) anos, tempo médio de formação de 7,93 (DP = 5,58) anos e tempo médio de experiência em TMO de 4,86 (DP = 2,38) anos. Todos os enfermeiros seniores possuíam, no mínimo, 36 meses de experiência como enfermeiro e Especialização em Oncologia.

A primeira fase, pré infusão do TCTH autólogo na modalidade criopreservada, compreendeu o intervalo entre 00:00 horas do dia zero até o início do período da tarde, momento em que ocorre o procedimento de contato com o Centro de Armazenamento Celular para confirmação do horário do início da infusão das CTH.

Indica-se, na Tabela 1, que dentre os 11 procedimentos (100,00%) realizados por enfermeiros seniores, na fase pré infusão do TCTH, os maiores CDM totais foram: Desinfecção e preparo do equipamento aquecedor de líquidos (US$ 25,55 – DP = 2,52), Preparo e administração de medicamentos endovenosos e subcutâneos (US$ 8,55 – DP = 3,57), Coleta de exames laboratoriais (US$ 5,50 – DP = 5,42, e Auxílio e realização de higiene corporal e limpeza concorrente do leito e mobiliários (US$ 5,28 – DP = 2,31). Considerando a somatória dos CDM totais dos 11 procedimentos, os quais resultaram em 718 observações, obtém-se o CDM total de US$59,52 correspondentes à fase pré infusão do TCTH autólogo na modalidade criopreservada. Na maioria dos procedimentos (90,90%), prevaleceu o CDM com a MOD de enfermeiros seniores na composição do CDM total.

**Tabela 1:** Distribuição dos custos em dólar (US$) com MOD, insumos (medicamentos/soluções e materiais) requeridos nos procedimentos realizados por enfermeiras seniores na fase pré infusão de TCTH Autólogo, criopreservado, segundo o custo direto médio (CDM), desvio padrão (DP), custo mínimo, custo máximo, 1º quartil, mediana e 3º quartil – São Paulo, SP, Brasil, 2025.

Procedimentos (número de observações)	CDM com MOD de enfermeiros US$*	CDM com insumos (medicamentos/soluções e materiais) US$*	CDM total US$*	Desvio-padrão US$*	Custo mínimo US$*	Custo máximo US$*	1º quartil US$*	Mediana US$*	3º quartil US$*
1. Coleta de exames laboratoriais (n = 19)	3,31	2,19	5,50	5,42	2,18	17,77	2,52	2,88	3,95
2. Aferição de sinais vitais, glicemia capilar e mensuração do peso corporal (n = 120)	0,97	0,05	1,02	0,46	0,38	2,96	0,74	0,93	1,29
3. Ronda intencional (n = 57)	0,77	0,00	0,77	0,28	0,37	1,66	0,55	0,74	0,92
4. Auxílio e realização de higiene corporal (banho) e limpeza concorrente do leito e mobiliários (n = 48)	3,42	1,86	5,28	2,31	1,88	9,10	3,13	4,38	7,22
5. Auxílio para eliminações vesico intestinais, refeições e controle hídrico (n = 85)	4,25	0,68	4,94	2,52	0,59	10,73	2,82	4,67	6,70
6. Teste de funcionamento do monitor, separação, organização e disposição dos insumos e equipamentos de emergência (n = 55)	2,62	0,00	2,62	1,81	0,55	7,01	1,29	1,84	3,69
7. Desinfecção e preparo do equipamento aquecedor de líquidos (n = 16)	10,90	14,64	25,55	2,52	20,47	28,58	23,92	25,96	27,59
8. Preparo e administração de medicamentos EV e SC (n = 82)	4,64	3,92	8,55	3,57	3,18	26,81	5,68	6,97	8,30
9. Preparo e administração de medicamentos VO (n = 145)	1,14	0,41	1,55	3,07	0,31	36,77	0,68	0,90	1,61
10. Troca de curativo de cateter venoso central, teste de fluxo e refluxo do cateter (n = 75)	2,49	0,62	3,09	1,15	1,31	5,82	2,04	2,76	3,28
11. Contato com o centro de armazenamento celular (n = 16)	0,65	0,00	0,65	0,34	0,18	1,29	0,37	0,65	0,78

*Taxa de conversão: R$ 5,75/US$ 1.00. Banco Central, com base na cotação média de fevereiro a abril de 2025.

Quanto ao consumo de materiais medicamentos e soluções, insulina regular (US$24,89), insulina NPH (US$8,63), água destilada 1000ml (US$13,10) e frasco anaeróbio (US$4,37) foram os de maiores custos unitários.

A segunda fase, intra infusão do TCTH, compreendeu o ato infusional, a monitorização contínua e o controle rigoroso dos sinais vitais, além da intervenção imediata do enfermeiro sênior, caso necessário devido a ocorrência de eventos adversos. Durante a condução do estudo, sete pacientes apresentaram reações adversas, sendo quatro, caracterizadas por febre e alterações sistêmicas, resultantes na coleta de exames laboratoriais, especificamente hemocultura, com um CDM de US$17,46. Entre os insumos utilizados, os frascos anaeróbios apresentaram um custo unitário elevado (US$52,38), correspondendo a 89,68% do CDM dos insumos consumidos. Mostra-se, na Tabela 2, que dos 10 procedimentos (100,00%) executados os de maiores CDM totais foram: Infusão da bolsa de CTH (US$631,62 – DP = 1,66) e Coleta de exames laboratoriais (US$17,46 – DP = 0,24). Considerando a somatória dos CDM totais desses 10 procedimentos, que resultaram em 375 observações, obteve-se o CDM total de US$663,45 (100,00%). Apesar do CDM com a MOD de enfermeiros predominar, na maioria dos procedimentos (70,00%), o CDM com insumos foi o de maior contribuição na composição do CDM total.

**Tabela 2:** Distribuição dos custos em dólar (US$*) com MOD, insumos (medicamentos/soluções e materiais) requeridos nos procedimentos realizados por enfermeiros seniores na fase intra infusão de CTH no TCTH Autólogo, criopreservado, segundo o custo direto médio (CDM), desvio padrão (DP), custo mínimo, custo máximo,1º quartil, mediana e 3º quartil e mediana – São Paulo, SP, Brasil, 2025.

Procedimentos (número de observações)	CDM com MOD de enfermeiros US$*	CDM com insumos (medicamentos/soluções e materiais) US$*	CDM total US$*	Desvio-padrão US$*	Custo mínimo US$*	Custo máximo US$*	1º quartil US$*	Mediana US$*	3º quartil US$*
1. Aferição de sinais vitais, glicemia capilar e monitorização do paciente (n = 165)	0,49	0,03	0,52	0,27	0,19	1,86	0,38	0,38	0,56
2. Orientações e esclarecimento sobre o procedimento da infusão de CTH (n = 19)	1,28	0,00	1,28	0,54	0,55	2,58	0,92	1,29	1,47
3. Encaminhamento e auxílio para eliminações vesico-intestinais, e controle hídrico (n = 14)	0,82	1,32	2,14	0,62	1,22	3,38	1,59	1,96	2,54
4. Limpeza concorrente dos mobiliários (n = 16)	1,06	0,78	1,85	0,39	1,13	2,42	1,49	1,69	1,90
5. Dupla checagem e descongelamento das bolsas de CTH (n = 90)	0,35	0,49	0,84	1,04	0,05	3,73	0,25	0,42	0,61
6. Infusão da bolsa de CTH (n = 45)	3,27	628,36	631,62	1,66	628,10	637,13	630,68	631,23	632,23
7. Preparo e administração de medicações (EV) (n = 4)	2,67	0,90	3,57	1,83	0,88	4,88	2,06	3,40	4,48
8. Coleta de exames laboratoriais (n = 4)	2,86	14,60	17,46	0,24	16,68	17,24	16,90	17,04	17,14
9. Lavagem do equipo pós infusão de CTH (n = 16)	1,91	0,84	2,76	0,68	1,01	3,77	1,51	1,84	2, 34
10. Troca de curativo de cateter venoso central, teste de fluxo e refluxo do cateter (n = 18)	0,97	0,45	1,41	0,35	0,37	2,11	1,21	1,36	1,41

*Taxa de conversão: R$ 5,75/US$ 1.00. Banco Central, com base na cotação média de fevereiro a abril de 2025.

Dentre os insumos consumidos na fase intra infusão de TCTH, bolsa de CTH (US$627,91), frasco anaeróbio (US$4,37) e comadre (US$1,10), foram os de maiores custos unitários.

A terceira fase, pós infusão do TCTH, abrangeu a observação sistemática dos procedimentos realizados após a lavagem do equipo, subsequente à infusão das CTH, contemplando todas as observações dos procedimentos executados no final do turno vespertino, estendendo-se até a meia noite do dia zero.

Na Tabela 3, verifica-se que dentre os sete procedimentos (100,00%) realizados os maiores CDM totais foram: Preparo e administração de medicamentos endovenosos e subcutâneos (US$6,77 – DP = 2,06), Limpeza concorrente dos mobiliários (US$2,40 – DP = 0,35) e Auxílio para eliminações vesico intestinais, refeições e controle hídrico (US$2,29 – DP = 1,08). Considerando a somatória dos CDM totais dos sete procedimentos, que resultaram em 463 observações, obteve-se o CDM total de US$16,84. Na maioria dos procedimentos (85,71%), prevaleceu o CDM com a MOD de enfermeiros seniores na composição do CDM total.

**Tabela 3:** Distribuição dos custos em dólar (US$) com MOD, insumos (medicamentos/soluções e materiais) requeridos nos procedimentos realizados por enfermeiros seniores na fase pós infusão de CTH no TCTH Autólogo, criopreservado, segundo o custo direto médio (CDM), desvio padrão (DP), custo mínimo, custo máximo e mediana – São Paulo, SP, Brasil, 2025.

Procedimentos (número de observações)	CDM com MOD de enfermeiros US$*	CDM com insumos (medicamentos/soluções e materiais) US$*	CDM total US$*	Desvio-padrão US$*	Custo mínimo US$*	Custo máximo US$*	1º quartil US$*	Mediana US$*	3º quartil US$*
1. Aferição de sinais vitais, glicemia capilar e mensuração do peso corporal (n = 176)	0,77	0,03	0,80	0,26	0,38	2,27	0,58	0,74	0,93
2. Limpeza concorrente dos mobiliários (n = 16)	1,11	1,30	2,40	0,35	1,65	2,81	1,99	2,20	2,55
3. Auxílio para eliminações vesico intestinais, refeições e controle hídrico (n = 77)	1,64	0,65	2,29	1,08	0,79	5,64	1,34	2,46	2,90
4. Esvaziamento e limpeza do equipamento aquecedor de líquidos (n = 16)	1,53	0,35	1,88	0,54	0,91	2,75	1,29	1,67	2,00
5. Preparo e administração de medicamentos endovenosos e subcutâneos (n = 55)	4,10	2,66	6,77	2,06	2,45	11,14	3,92	5,42	7,12
6. Preparo e administração de medicamentos via oral (n = 110)	0,81	0,17	0,98	0,73	0,28	3,07	0,49	0,67	1,01
7. Troca de curativo de cateter venoso central, teste de fluxo e refluxo do cateter (n = 13)	1,09	0,62	1,72	1,06	0,75	3,77	0,93	1,12	1,32

*Taxa de conversão: R$ 5,75/US$ 1.00. Banco Central, com base na cotação média de fevereiro a abril de 2025.

Na fase pós infusão de TCTH, os insumos insulina regular (US$24,89), água destilada 1000ml (US$13,10) e insulina NPH (US$8,63) foram os de maiores custos unitários. O equipo macrogotas com injetor lateral e filtro de ar (US$2,81), foi o material mais utilizado (42,19%) na administração das soluções de hidratação.

## DISCUSSÃO

Todos os procedimentos realizados no “dia zero” do TCTH foram executados exclusivamente por enfermeiros seniores com competência clínica especializada comprovada, evidenciando o compromisso do HPT em manter equipe altamente qualificada, visando a realização segura das etapas essenciais do TCTH. Destaca-se a imprescindibilidade da atuação de enfermeiros, devidamente capacitados, nesse momento crítico do transplante, visto que o sucesso do procedimento, a segurança do paciente e a prevenção ou minimização de eventos adversos dependem diretamente da expertise técnica desses profissionais.

O enfermeiro desempenha papel central no cuidado ao paciente submetido ao TCTH, sua expertise técnica é determinante para a segurança, prevenção de complicações e condução qualificada dos procedimentos requeridos^(16,17)^.

Na fase pré infusão, 59,07% do CDM total correspondeu a MOD dos enfermeiros seniores; os custos mais expressivos (US$25,55) foram decorrentes da desinfecção e preparo do equipamento aquecedor de líquidos. Esclarece-se, no que diz respeito às práticas da infusão de CTH criopreservadas, que o descongelamento das bolsas exige equipamento hospitalar e técnicas cuidadosamente validadas (controle rigoroso da temperatura, limpeza e desinfecção adequadas e técnica estéril), para garantir a viabilidade celular, minimizar os riscos de contaminação e garantir a segurança do receptor^(18)^.

A administração de medicamentos endovenosos e subcutâneos (CDM = US$ 8,55) se destacou na composição do CDM total da fase pré infusão, sendo um procedimento necessário para o manejo clínico, por intermédio de medidas farmacológicas voltadas à prevenção de eventos adversos (EA) relacionados à infusão.

Estudo que analisou 1.269 infusões de CTH autólogas criopreservadas, contendo bolsas com o reagente crioprotetor dimetilsufóxico (DMSO), em 1.191 pacientes adultos, verificou que 37,8% das infusões apresentaram algum EA. O DMSO foi associado à maioria das reações, reforçando que intervenções clínicas, especialmente ajustes e administração de medicamentos pré infusão, podem reduzir a toxicidade e aumentar a segurança do transplante. A frequência desses EA, classificados como leves ou moderados, indica a pertinência de estratégias preventivas, como exemplos hidratação adequada, uso de anti-histamínicos e antieméticos, capazes de mitigar riscos e aprimorar a segurança da infusão de CTH^(19)^. Entretanto, dentre os medicamentos utilizados neste estudo, a insulina regular (US$ 24,89) apresentou o maior CDM, em razão de comorbidades pré-existentes que requereram ajustes glicêmicos para o manejo clínico seguro dos pacientes transplantados.

Os CDM da coleta de exames laboratoriais (US$ 5,50); auxílio e realização de higiene corporal e limpeza concorrente do leito e mobiliário (US$ 5,28), também repercutiram no CDM total da fase pré infusão. Revisão sistemática^(20)^, abrangendo estudos realizados em unidades de transplante de células hematopoiéticas, demonstrou que diversos patógenos estavam diretamente associados a fonte ambientais, surtos virais e práticas inadequadas de desinfecção. Esses achados indicam as boas práticas de higiene, limpeza e desinfecção como um investimento essencial para evitar infecções que podem resultar em piora clínica, prolongamento de internações e aumentar, significativamente, os custos assistenciais^(20)^.

Dentre os procedimentos executados na fase intra infusão, a infusão da bolsa de CTH (95,20%), apresentou a maior contribuição à composição do CDM total (US$663,45 – 100,00%), seguida da coleta de exames laboratoriais (2,63%).

Um estudo^(21)^ que analisou o ciclo completo de coleta, criopreservação, armazenamento e utilização de CTH em um centro transplantador europeu, ao longo de 12 anos, evidenciou que a coleta, processamento, armazenamento e descarte de CTH, envolve custos substanciais e frequentemente subestimados, especialmente quando consideradas as bolsas que nunca chegam a ser utilizadas em transplantes. Verificou que a infusão de CTH ocorre majoritariamente em curto intervalo após a coleta, e que o manejo das bolsas armazenadas por longos períodos resulta em dispêndios elevados para os serviços^(21)^.

Outro estudo^(22)^, que avaliou 613 pacientes com mieloma múltiplo por nove anos, constatou que a taxa de utilização das bolsas armazenadas por mais de 30 dias foi extremamente baixa (14,9%), e que 69,00% dos pacientes mantiveram bolsas de CTH criopreservadas estocadas, por dois anos ou mais, com custos expressivos associados à manutenção em nitrogênio líquido, procedimentos adicionais de aférese e descarte tardio. Grande parte dos custos relacionados ao ciclo das CTH não se concentra no momento da infusão, mas nas etapas anteriores de mobilização, coleta e armazenamento prolongado, processos padronizados e que influenciam, diretamente, o valor final dos insumos utilizados no momento da administração à beira-leito^(22)^.

Ambos os estudos^(21,22)^ supracitados, destacam que as instituições de saúde necessitam revisar suas práticas e fluxos relacionados à gestão das CTH para otimizar recursos, reduzir desperdícios e garantir sustentabilidade econômico-operacional do TCTH.

Na fase intra infusão das CTH, a ocorrência de reações adversas requereu a coleta de exames laboratoriais e os frascos anaeróbios para hemocultura corresponderam a itens de custo unitário elevado. Estudo de revisão narrativa destacou que a maior parte das hemoculturas coletadas em ambiente hospitalar, com indicações baseadas em febre ou alterações inespecíficas, apresentam baixa correlação com contaminação bacteriana e enfatizou que a coleta indiscriminada contribui para a elevação dos custos^(23)^. Assim, embora a investigação de febre durante o ato infusional de CTH seja clinicamente pertinente, a indicação criteriosa da hemocultura torna-se essencial para equilibrar acurácia diagnóstica, segurança do paciente e racionalidade no uso de insumos, particularmente aqueles de elevado custo unitário.

Na fase pós infusão das CTH, o preparo e a administração de medicamentos (US$ 6,77 – 40,20%) correspondeu ao maior CDM. O material mais utilizado, na administração das soluções de hidratação, foi o equipo macrogotas com injetor lateral e filtro de ar (US$2,81) juntamente com o soro fisiológico 0,9% 1000 mL (US$1,07). Isso porque uma boa prática adotada na UTCTH consiste na substituição do equipo a cada preparo de solução hidratante, associada ao uso de bomba de infusão contínua. Justifica-se a ênfase na hidratação após a infusão das CTH criopreservadas em DMSO, estudo de revisão narrativa descreveu que a infusão das CTH com DMSO está relacionada a efeitos nefrotóxicos, cardiovasculares, neurológicos e gastrintestinais^(24)^.

Estudo^(25)^ investigou a variação das práticas de hidratação intravenosa, após a infusão de CTH autólogas criopreservadas em DMSO, em 510 centros transplantadores vinculados ao *European Bone Marrow Transplantation*. Os resultados demonstraram que a hidratação pós infusão é amplamente utilizada para proteger a função renal e auxiliar na eliminação do DMSO do organismo; contudo, ainda não há evidências científicas que confirmem sua eficácia na redução da toxicidade associada^(25)^.

O segundo procedimento com maior CDM na fase pós infusão foi a limpeza concorrente dos mobiliários (US$ 2,40). Esse valor é inferior ao obtido na fase pré infusão das CTH (US$ 5,28), visto que, nela o custo não envolve o encaminhamento e auxílio para a realização da higiene corporal do paciente.

Apesar do CDM com insumos ter sido o de maior contribuição na composição do CDM total da fase intra TCTH, nas três fases prevaleceu o CDM com a MOD dos enfermeiros seniores. Estudos^(17,18,19,20,21,22,23,24,25,26)^ reforçam que o enfermeiro é o profissional que atua mais proximamente ao paciente durante todas as fases do TCTH, o qual tem elevada complexidade e é permeado por intensa carga emocional e alto risco clínico, desempenhando papéis que incluem vigilância clínica, manejo de efeitos adversos, apoio emocional e educação terapêutica que requerem elevada expertise profissional^(17,18,19,20,21,22,23,24,25,26)^. Tais atividades, que também foram observadas neste estudo, justificam por que a MOD do enfermeiro representou a maior parcela do CDM.

Evidencia-se que o cuidado de enfermagem em TCTH tem impacto direto na prevenção de complicações, especialmente por meio da vigilância clínica sistemática, da implementação de protocolos de segurança e do monitoramento precoce de efeitos toxicológicos e infecciosos demonstram que a padronização de procedimentos de enfermagem na infusão autóloga de células-tronco reduz variabilidade assistencial e contribui para resultados clínicos mais seguros^(6)^. A presença contínua, a vigilância sistematizada, a execução de cuidados essenciais e a prevenção de EA situam os enfermeiros no núcleo operacional e assistencial dos cuidados requeridos no TCTH^(6,16,26,27)^. Ressalta-se que os custos associados à atuação dos enfermeiros não devem ser interpretados como um marcador de onerosidade, mas sim como um investimento essencial à segurança do paciente e qualidade assistencial. Dessa forma, os achados deste estudo corroboram a evidência de que o tempo despendido pela enfermagem em cuidados diretos é um componente indispensável para a segurança do TCTH.

### Implicações para a Prática

Conhecer os custos relacionados à assistência de enfermagem nas diferentes fases/etapas do dia zero do TCTH, notadamente em instituições públicas de saúde no contexto do Sistema Único de Saúde, pode auxiliar na formulação de estratégias abrangendo, também, os aspectos financeiros, privilegiando a revisão de processos e a alocação racional de recursos finitos, sem comprometimento da qualidade desejada, resultando em melhores desfechos para os pacientes.

### Limitação do Estudo

Indica-se, como limitação, a observação não participante restrita aos procedimentos relacionados ao TCTH autólogo na modalidade criopreservada, o que não permite a aplicação dos resultados obtidos para outros tipos de TCTH ou modalidades de infusão.

## CONCLUSÃO

O CDM total da assistência prestada por enfermeiras seniores à pacientes no dia zero do TCTH, a partir de 1556 observações de 28 procedimentos, correspondeu a US$739,81 (100,00%) sendo 8,04% relativos à fase pré TCTH, 89,68% à fase intra TCTH e 2,28% à fase pós TCTH. Nas três fases, houve o predomínio do CDM com a MOD dos enfermeiros na composição do CDM total. Contudo, na fase intra TCTH, o CDM com insumos foi o de maior contribuição na composição do CDM total, destacando-se a bolsa de CTH (valor unitário de US$627,91).

## DISPONIBILIDADE DE DADOS

Não se aplica. Trata-se de um estudo de caso único cujo conjunto de dados que dá suporte aos resultados não está disponível publicamente devido à presença de informações sensíveis e à necessidade de preservação da confidencialidade dos participantes. Todas as informações relevantes para a compreensão dos achados estão descritas no corpo do artigo.

# Translation: Día cero del trasplante autólogo de células madre hematopoyéticas criopreservadas: microcosteo de la asistencia prestada por enfermeros senior*

**DOI:** 10.1590/1980-220X-REEUSP-2025-0528es

**Authors:** Valéria Aparecida Gomes Nunes, Antônio Fernandes Costa Lima

**Affiliations:** 1 Universidade de São Paulo, Escola de Enfermagem, Programa de Pós-Graduação em Gerenciamento em Enfermagem, São Paulo, SP, Brasil.; 2 Universidade de São Paulo, Escola de Enfermagem, Departamento de Orientação Profissional, São Paulo, SP, Brasil.

**Keywords:** Trasplante de células madre hematopoyéticas, Trasplantados, Asistencia hospitalaria, Cuidados de enfermería, Costos directos de los servicios

## Abstract

**Objetivo:**

Medir el costo directo promedio de la asistencia prestada por enfermeros senior a pacientes en el día cero del trasplante autólogo de células madre hematopoyéticas criopreservadas en un hospital público de trasplantes del estado de São Paulo.

**Método:**

Investigación cuantitativa, exploratoria-descriptiva, de tipo estudio de caso único, basada en el microcosteo ascendente, realizada entre febrero y abril de 2025.

**Resultados:**

Se observaron 28 procedimientos realizados por enfermeros seniores a 16 pacientes el día cero del trasplante, abarcando las fases previas (n = 718), intra (n = 375) y posteriores a la infusión (n = 463) de células madre hematopoyéticas. En la fase previa a la infusión del trasplante de células madre hematopoyéticas se obtuvo un costo directo medio total de 59,52 dólares estadounidenses; en la fase intrainfusión, 663,45 dólares estadounidenses, y en la fase posterior a la infusión, 16,84 dólares estadounidenses. El costo directo medio predominante en las tres fases fue el de la mano de obra de los enfermeros. Sin embargo, en la fase intrainfusión, el costo de los insumos fue el que más contribuyó a la composición del costo directo medio total.

**Conclusión:**

Teniendo en cuenta las tres fases (n = 1556 observaciones), el costo directo medio total fue de 739,81 dólares estadounidenses (100,00%), de los cuales el 8,04% correspondió a la fase previa a la infusión, el 89,68% a la fase intrainfusión y el 2,28% a la fase postinfusión.

## INTRODUCCIÓN

Brasil se consolida mundialmente como el segundo país en número de trasplantes. Es reconocido por tener el programa público de trasplantes de órganos y tejidos más completo del mundo, financiado en su mayor parte con recursos públicos por el Sistema Único de Salud (SUS)^(1,2)^. Según el Registro Brasileño de Trasplantes de la Asociación Brasileña de Trasplantes de Órganos y Tejidos, en 2024 se realizaron 26 509 trasplantes de órganos y 3821 trasplantes de células madre hematopoyéticas (TCMH). De los TCMH, 2,345 fueron trasplantes autólogos y 1,476 alogénicos. Del total de TCMH, 1,664 se realizaron en la ciudad de São Paulo, de los cuales 887 fueron trasplantes autólogos y 777 alogénicos^(3)^.

El TCMH consiste en un procedimiento terapéutico electivo de alta complejidad, potencialmente curativo, capaz de reconstituir el sistema hematopoyético e inmunológico en pacientes con enfermedades hematológicas malignas y no malignas, autoinmunes, congénitas y metabólicas^(4)^. Se realiza después de la supresión medular, mediante la administración de dosis de quimioterapia o radioterapia, con la sustitución de las células insalubres de la médula ósea (MO) por células madre hematopoyéticas (CMH) sanas para restablecer la hematopoyesis. Requiere vigilancia y manejo especializado tanto para las complicaciones inmediatas como para las tardías que pueden ocurrir a largo plazo.

La MO se consideraba la principal fuente de recolección de CMH, sin embargo, los avances científicos han permitido el uso de fuentes alternativas, como la sangre periférica y la sangre del cordón umbilical y placentario. Las CMH pueden obtenerse de dos fuentes principales: autólogas, procedentes de la MO y de la sangre periférica cuando se extraen del propio paciente, o alogénicas, extraídas de la MO, la sangre periférica y la sangre del cordón umbilical placentario de un donante, que puede estar relacionado o no con la familia del paciente^(4)^.

Según la Red Mundial de Trasplantes de Sangre y Médula Ósea, la implementación de un servicio de TCMH debe llevarse a cabo de manera estructurada, progresiva y orientada por los principios de calidad y seguridad del paciente y del donante.

Los requisitos para el establecimiento de programas de TCMH autólogo y alogénico pueden variar en función del tipo de trasplante, las indicaciones, la disponibilidad de recursos y el nivel de complejidad que implique. Entre los elementos imprescindibles para la viabilidad de estos programas destacan: una infraestructura adecuada, que debe incluir instalaciones para la realización de aféresis, un laboratorio de procesamiento celular o acceso a servicios de laboratorio para el recuento de células, la crioconservación y el almacenamiento de las CMH, así como un área de aislamiento para los pacientes y un equipo multidisciplinario especializado en hematología, manejo de quimioterapia, control de infecciones hospitalarias y cuidados intensivos que garantice la seguridad y eficacia del procedimiento^(5)^.

El «día cero» corresponde al momento de la infusión de las CMH y es la etapa principal del TCMH, que requiere protocolos institucionales basados en evidencia científica, lo cual es determinante para reducir riesgos, calificar la asistencia, promover la seguridad del paciente y favorecer el éxito terapéutico del TCMH^(6)^.

El enfermero es responsable de infundir las CMH y garantizar una serie de cuidados esenciales, incluyendo la preparación y administración de medicamentos antes, durante y después de la infusión; la monitorización rigurosa y la vigilancia constante con el fin de prevenir, identificar e intervenir en las complicaciones relacionadas con el procedimiento^(6)^, lo que genera costos que deben identificarse y gestionarse adecuadamente.

En las organizaciones de salud, los enfermeros son fundamentales en la gestión de los recursos financieros y asistenciales; debido a su actuación ininterrumpida las 24 horas del día, tienen un conocimiento detallado de las necesidades de los pacientes y de los recursos necesarios para atender las demandas presentadas, lo que les capacita para actuar en el análisis y control de los costos asociados^(7)^.

Un estudio de análisis económico correlacionó la recopilación de datos de 4513 TCMH, realizados en 2017, en 13 de los 28 países pertenecientes al Latin America Bone Marrow Transplant Group (México, Cuba, Panamá, Costa Rica, Colombia, Perú, Brasil, Ecuador, Venezuela, Uruguay, Paraguay, Chile y Argentina). Se observó una variación de 12 TCMH realizados en Venezuela a 345 TCMH en Uruguay; las tasas de trasplantes fueron más altas en los países donde los procedimientos costaban más, cuando se realizaban en hospitales privados con fines de lucro y se pagaban con fondos públicos. Los costos de los TCMH autólogos variaron de 65 000 dólares estadounidenses en Costa Rica a 900 000 dólares estadounidenses en Panamá; y los costos de un TCMH alogénico de un familiar variaron de 150 000 dólares estadounidenses en México a 860 000 dólares estadounidenses en Uruguay. Se destacó la complejidad de estimar con precisión el costo del TCMH en los hospitales públicos, ya que no se consideraba el costo de la mano de obra del personal médico y de enfermería, ni los costos de muchos medicamentos ampliamente utilizados, como antibióticos, fluidos intravenosos, insumos desechables, entre otros, que normalmente se tienen en cuenta en los hospitales privados^(8)^.

Conocer los costos asociados a las diferentes fases del TCMH, especialmente en las instituciones públicas de salud en el contexto del SUS, puede ayudar a formular estrategias de tratamiento, privilegiando la asignación racional de recursos finitos, sin comprometer la calidad deseada, lo que se traduce en mejores resultados para los pacientes trasplantados.

En esta perspectiva, el uso del microcosteo ascendente se ha extendido en el área de la salud, ya que subsidia la identificación y valoración directa de cada recurso consumido, artículo por artículo (tiempo dedicado por el profesional, consumo de medicamentos y materiales), para cada paciente o servicio específico. Esta información ayuda a la asignación eficiente de recursos, a la planificación presupuestaria y a la definición de prioridades, contribuyendo a la sostenibilidad del sistema de salud. Este método es útil para determinar los costos de nuevas tecnologías, tratamientos o intervenciones; proporciona datos sólidos y basados en la evidencia para fundamentar la toma de decisiones de los gerentes, administradores y formuladores de políticas de salud^(9,10,11)^.

Teniendo en cuenta que la TCMH es un procedimiento de alta complejidad, elevado costo e impacto significativo en los sistemas de salud, especialmente en los países en desarrollo, se observa una importante desigualdad en el acceso a esta terapia. A pesar del crecimiento progresivo del número de TCMH en las últimas décadas en América Latina, las tasas de realización siguen siendo entre siete y ocho veces inferiores en comparación con las tasas observadas en Estados Unidos y Europa, que presentan una variación de hasta 30 veces entre los países de la propia región, debido a factores socioeconómicos, estructurales y de financiación del sistema de salud^(8)^. Además, aún son escasos los estudios que miden, de forma objetiva y detallada, los costos directos asociados a las etapas críticas del TCMH, en particular los relacionados con la asistencia de enfermería, cuyos profesionales desempeñan un papel fundamental en el mantenimiento de la seguridad y la calidad de la atención prestada.

Por lo tanto, este estudio tiene como objetivo medir el costo directo medio (CDM) de la asistencia prestada por enfermeros senior a pacientes en el día cero del trasplante autólogo de células madre hematopoyéticas (TCMH) criopreservadas, en un hospital público de trasplantes (HPT) del estado de São Paulo.

## MÉTODO

### Tipo o Diseño del Estudio

Se trata de una investigación cuantitativa, exploratoria/descriptiva, del tipo estudio de caso único.

### Lugar

El HPT, vinculado a la Secretaría de Salud del Estado de São Paulo y gestionado por una Organización Social de Salud, es una referencia en diferentes especialidades, destacando en las áreas de oncohematología, hemofilia, urología, oftalmología, nefrología y neurocirugía. Su actuación se basa en la mejora continua de la asistencia, la calidad y la seguridad del paciente y la gestión eficiente de los procesos organizativos. Presta atención exclusiva a través del Sistema Único de Salud (SUS), garantizando el acceso regulado en función de criterios técnicos y de justicia distributiva en el proceso de derivación de los pacientes referidos, realizado de manera equitativa, ordenada, oportuna y racional por la Central de Oferta y Regulación de Servicios de Salud, con la disponibilidad y asignación de plazas operativas a través del Sistema Informatizado de Regulación del Estado de São Paulo.

El HPT, con experiencia previa en la estructuración de líneas de atención asistencial, consolidó, a partir de 2022, una línea de atención dirigida específicamente al TCMH, autólogo o alogénico, que abarca, de forma continua e integrada, todas las fases de la atención multiprofesional, desde la consulta previa al TCMH, la hospitalización para la realización del TCMH, hasta la atención ambulatoria posterior al TCMH.

La Unidad de Hospitalización de Trasplante de Células Madre Hematopoyéticas (UTCMH) cuenta con 16 camas distribuidas en 14 habitaciones, 12 de ellas con cama individual y baño privado y dos con dos camas y un baño colectivo. Dispone de aire acondicionado, con control de temperatura entre 18 y 21 °C, y estructura de aire ambiente protector con presión positiva y filtro de alta eficiencia para la retención de partículas, para la prevención de infecciones fúngicas, y buenas prácticas para actividades con CMH para uso terapéutico en TCMH, cumpliendo íntegramente los requisitos de la RDC n.º 508/2021 de la Agencia Nacional de Vigilancia Sanitaria^(12)^. La hospitalización es individualizada y se basa en la atención centrada en el paciente, y puede abarcar desde la movilización, la Recolección, el procesamiento, el acondicionamiento, la infusión de CMH, la pancitopenia, el injerto leucocitario y el monitoreo de complicaciones hasta el alta, lo que comprende el plan terapéutico.

Debido a la complejidad y especificidad de la atención de la UTCMH, en ella solo trabajan enfermeros senior (contratados con un mínimo de 36 meses de experiencia como enfermeros y con especialización en el área de actuación prevista, en régimen de Consolidación de las Leyes del Trabajo), con una carga de trabajo de 36 horas semanales. Cada uno de los turnos de trabajo (mañana, tarde, noche par e impar) cuenta con siete enfermeros senior, lo que suma un total de 28.

En 2024, se realizaron 121 TCMH, de los cuales 110 (90,9%) correspondieron a la modalidad autóloga y 11 (9,1%) a la modalidad alogénica. La distribución mensual de estos procedimientos varió de 1 a 15 TCMH, con un promedio de 10,8 TCMH mensuales. De enero a septiembre de 2025, se realizaron 81 TCMH, de los cuales 72 (88,9%) fueron autólogos y 9 (11,1%) alogénicos, con una variación mensual de 1 a 16 TCMH y un promedio de 9 TCMH mensuales.

### Muestra y Criterios de Selección

Se estableció un muestreo intencional no probabilístico con la observación no participante de, como mínimo, 10,8 TCMH (promedio mensual de pacientes trasplantados en 2024), en la modalidad autóloga criopreservada, abarcando tres meses típicos de atención de la UTCMH. Se incluyeron los procedimientos que forman parte de la atención prestada por enfermeros senior a los pacientes el día cero del TCMH autólogo, en el periodo comprendido entre febrero y abril de 2025.

### Protocolo del Estudio

Previo a la aprobación del Comité de Ética en Investigación (CEP) del HPT, se obtuvo el consentimiento del Director y del Gerente de Enfermería de la institución. Tras la aprobación del CEP, se mapearon, a partir de los expedientes electrónicos de los pacientes sometidos a TCMH en la modalidad autóloga criopreservada, considerando tres meses típicos de funcionamiento, los procedimientos realizados por enfermeros senior el día cero, así como los insumos (materiales, medicamentos y soluciones) necesarios para su viabilidad.

Una de las autoras, que es supervisora de enfermería desde hace siete años en el HPT, llevó a cabo una revisión de la rutina institucional, junto con dos enfermeros con amplia experiencia en TMO, con el fin de garantizar la estandarización de la asistencia prestada por el enfermero senior en el día cero del TCMH.

El documento denominado «Rutina de la Unidad de Hospitalización – TMO: Procedimientos en el día cero del Trasplante de Células Madre Hematopoyéticas en la modalidad criopreservada» fue validado por los enfermeros senior, analizado y aprobado por las Direcciones Administrativa, Médica y de Enfermería del HPT. Posteriormente, se puso a disposición para su consulta en el Sistema de Gestión Integrada (SOGI) institucional y se favoreció la capacitación de los enfermeros senior que trabajan en los tres turnos de la UTCMH, en cuanto a los cambios/ajustes requeridos para su implementación.

Tras constatar que todos los enfermeros senior cumplían adecuadamente con la rutina establecida, se propuso un instrumento para la recolección de datos dividido en: 1) Caracterización del receptor de TCMH criopreservadas en la modalidad autóloga; 2) Datos de la infusión de células madre: día cero; 3) Costo Directo Medio (CDM) de los procedimientos realizados el día cero del TCMH con la infusión de CMH criopreservadas en la modalidad autóloga; que también fue evaluado, modificado con la inclusión de sugerencias y validado por los enfermeros senior. Todos los enfermeros senior aceptaron la invitación para participar en el estudio. Tras la firma del Término de Consentimiento Libre e Informado, la autora Supervisora de Enfermería procedió a la recolección de datos, de febrero a abril de 2025, incluyendo las observaciones no participantes de las TCMH criopreservadas en modalidad autóloga.

El estudio se basó en los costos directos, que se refieren a todos aquellos identificables y medibles, relacionados con un procedimiento determinado, que pueden atribuirse a un centro de costos correspondiente, como los insumos hospitalarios tales como: agujas, jeringas, sondas, medicamentos y mano de obra directa (MOD) de recursos humanos específicos^(13)^.

Se utilizó el método de microcosteo ascendente (bottom-up) por permitir definir, en detalle, las variables que componen los costos, a partir de datos individuales del tratamiento del paciente, con la revisión de expedientes médicos o fichas clínicas específicas para el estudio^(10,11)^.

La MOD se refiere a los profesionales que trabajan directamente en un determinado producto o servicio, cuando es posible medir el tiempo dedicado a su realización. Se compone del salario, las cargas sociales, las vacaciones y la paga extra^(13)^.

Se calculó el promedio de la masa salarial de la MOD de los 28 enfermeros senior a partir de los salarios medios (salarios, prestaciones, gratificaciones y cargas sociales) relativos a cuatro meses típicos de trabajo en la UITCMH, facilitados por el Departamento de Gestión de Personal. Así, se obtuvieron los valores de 9162,02 R$ por 144 horas, 63,63 R$ por hora y 1,06 R$ por minuto. Los costos de los materiales y medicamentos/soluciones fueron proporcionados por los Sectores de Compras, Farmacia y Almacén del HPT.

El CDM de los procedimientos realizados por enfermeros senior el día cero del TCMH autólogo criopreservado se obtuvo multiplicando el tiempo empleado (cronometrado) por el costo unitario de la MOD y sumándolo al costo de los insumos utilizados^(14)^.

Se utilizó la ecuación $\bar{C\left(P_{t}\right)}=\sum_{k=1}^{n}\left({\bar{q}}_{k}\cdot \bar{Pu_{k}}\right)+\sum_{k=1}^{n}\left({\bar{qS}}_{k}\cdot \bar{PSu_{k}}\right)+\sum_{c=1}^{n}\left(({\bar{t}}_{c}\cdot \bar{Su_{c}}\right)$ ; definiendo la cantidad media de materiales $\left[\bar{qm_{k}}\right]$ ; el precio unitario medio de cada material $\left[\bar{Pmu_{k}}\right]$ ; la cantidad media de soluciones/medicamentos $\left[\bar{qS_{k}}\right]$ ; el precio unitario medio de cada solución/medicamento $\left[\bar{PSu_{k}}\right]$ ; el tiempo medio de dedicación de cada categoría profesional $\left[\bar{t_{c}}\right]$ y la masa salarial unitaria media de la MOD de cada categoría profesional $\left[\bar{Su_{c}}\right]$ ^(14)^.

Se utilizó la moneda brasileña real (R$), posteriormente convertida a dólares estadounidenses (USD) al tipo de cambio medio de R$ 5,75/USD 1,00, basado en las cotizaciones de febrero a abril de 2025, proporcionadas por el Banco Central de Brasil.

### Análisis y Tratamiento de los Datos

Los datos se sistematizaron en hojas de cálculo electrónicas y posteriormente se transfirieron al Simulador de microcostos bottom-up^(15)^, desarrollado por el Grupo de Investigación Dimensión Económica de la Gestión en Enfermería. A continuación, se trataron mediante estadísticas descriptivas de posición (media, mínimo, máximo) y de escala (desviación estándar – DE).

### Aspectos Éticos

El CEP de la institución proponente aprobó la realización del estudio mediante el dictamen fundamentado n.º: 6.530.539, de 24/11/2023.

## RESULTADOS

De febrero a abril de 2025, se observó a 28 enfermeros senior durante la asistencia prestada a 16 (100,00%) pacientes sometidos a TCMH autólogo, en la modalidad criopreservada, en la UTCMH. Predominaron los pacientes de sexo masculino (56.25%), con mieloma múltiple (75.00%), edad media de 58.63 (DE = 11.24) años y que se autodeclararon de raza blanca (68.75%).

Entre los 28 (100,00%) enfermeros senior, predominaron las mujeres (92,86%), con una edad media de 36,46 (DE = 7,78) años, una formación media de 7,93 (DE = 5,58) años y una experiencia media en TMO de 4,86 (DE = 2,38) años. Todos los enfermeros sénior tenían, como mínimo, 36 meses de experiencia como enfermeros y especialización en oncología.

La primera fase, previa a la infusión del TCMH autólogo en modalidad criopreservada, abarcó el intervalo entre las 00:00 horas del día cero y el comienzo de la tarde, momento en el que se produce el contacto con el Centro de Almacenamiento Celular para confirmar la hora de inicio de la infusión de las CMH.

En la Tabla 1 se indica que, de los 11 procedimientos (100,00%) realizados por enfermeros senior en la fase previa a la infusión de TCMH, los mayores CDM totales fueron: Desinfección y preparación del equipo calentador de líquidos (25,55 dólares estadounidenses – DE = 2,52), Preparación y administración de medicamentos intravenosos y subcutáneos (8,55 dólares estadounidenses – DE = 3,57), Recolección de pruebas de laboratorio (5,50 dólares estadounidenses – DE = 5,42, y Ayuda y realización de la higiene corporal y limpieza concurrente de la cama y el mobiliario (5,28 dólares estadounidenses – DE = 2,31). Teniendo en cuenta la suma de los CDM totales de los 11 procedimientos, que dieron lugar a 718 observaciones, se obtiene un CDM total de 59,52 dólares correspondientes a la fase previa a la infusión del TCMH autólogo en la modalidad criopreservada. En la mayoría de los procedimientos (90,90%), prevaleció el CDM con la MOD de enfermeros superiores en la composición del CDM total.

**Tabla 1:** Distribución de los costes en dólares estadounidenses (USD) con MOD, insumos (medicamentos/soluciones y materiales) necesarios en los procedimientos realizados por enfermeros superiores en la fase previa a la infusión del TCMH autólogo criopreservado, según el coste directo medio (CMD), la desviación estándar (DE), el coste mínimo, el coste máximo, el primer cuartil, la mediana y el tercer cuartil – São Paulo, SP, Brasil, 2025.

Procedimientos (número de observaciones)	CDM con MOD de enfermeros USD*	CDM con suministros (medicamentos/soluciones y materiales) USD*	Total CDM USD*	Desviación estándar USD*	Coste mínimo USD*	Coste máximo USD*	1.er cuartil USD*	Mediana USD*	Tercer cuartil USD*
1. Recopilación de pruebas de laboratorio (n = 19)	3,31	2,19	5,50	5,42	2,18	17,77	2,52	2,88	3,95
2. Medición de constantes vitales, glucosa capilar y peso corporal (n = 120)	0,97	0,05	1,02	0,46	0,38	2,96	0,74	0,93	1,29
3. Disparo intencionado (n = 57)	0,77	0,00	0,77	0,28	0,37	1,66	0,55	0,74	0,92
4. Asistencia y realización de tareas de higiene personal (baño) y limpieza de la cama y los muebles (n = 48)	3,42	1,86	5,28	2,31	1,88	9,10	3,13	4,38	7,22
5. Asistencia con la micción y la defecación, las comidas y la ingesta de líquidos (n = 85)	4,25	0,68	4,94	2,52	0,59	10,73	2,82	4,67	6,70
6. Comprobación del monitor, separación, organización y disposición de los suministros y equipos de emergencia (n = 55)	2,62	0,00	2,62	1,81	0,55	7,01	1,29	1,84	3,69
7. Desinfección y preparación de equipos de calentamiento de líquidos (n = 16)	10,90	14,64	25,55	2,52	20,47	28,58	23,92	25,96	27,59
8. Preparación y administración de medicamentos intravenosos y subcutáneos (n = 82)	4,64	3,92	8,55	3,57	3,18	26,81	5,68	6,97	8,30
9. Preparación y administración de medicamentos orales (n = 145)	1,14	0,41	1,55	3,07	0,31	36,77	0,68	0,90	1,61
10. Cambio de apósito de catéter venoso central, prueba de flujo y reflujo del catéter (n = 75)	2,49	0,62	3,09	1,15	1,31	5,82	2,04	2,76	3,28
11. Contacto con el centro de almacenamiento celular (n = 16)	0,65	0,00	0,65	0,34	0,18	1,29	0,37	0,65	0,78

*Tipo de cambio: 5,75 R$/1,00 USD. Banco Central, basado en el tipo de cambio medio de febrero a abril de 2025.

En cuanto al consumo de materiales médicos y soluciones, la insulina regular (24,89 dólares estadounidenses), la insulina NPH (8,63 dólares estadounidenses), el agua destilada de 1000 ml (13,10 dólares estadounidenses) y el frasco anaeróbico (4,37 dólares estadounidenses) fueron los que tuvieron los mayores costos unitarios.

La segunda fase, la infusión de CMH, comprendió la infusión, la monitorización continua y el control riguroso de los signos vitales, además de la intervención inmediata del enfermero superior, en caso necesario debido a la aparición de eventos adversos. Durante la realización del estudio, siete pacientes presentaron reacciones adversas, cuatro de ellas caracterizadas por fiebre y alteraciones sistémicas, lo que dio lugar a la recolección de exámenes de laboratorio, concretamente hemocultivos, con un CDM de 17,46 dólares. Entre los insumos utilizados, los frascos anaeróbicos presentaron un costo unitario elevado (52,38 dólares), lo que corresponde al 89,68% del CDM de los insumos consumidos. En la Tabla 2 se muestra que, de los 10 procedimientos (100,00%) realizados, los que tuvieron un CDM total más alto fueron: Infusión de la bolsa de CMH (631,62 dólares estadounidenses – DE = 1,66) y Recolección de exámenes de laboratorio (17,46 dólares estadounidenses – DE = 0,24). Teniendo en cuenta la suma de los CDM totales de estos 10 procedimientos, que dieron lugar a 375 observaciones, se obtuvo un CDM total de 663,45 dólares (100,00%). A pesar de que el CDM con MOD de enfermeros predomina en la mayoría de los procedimientos (70,00%), el CDM con insumos fue el que más contribuyó a la composición del CDM total.

**Tabla 2:** Distribución de los costes en dólares estadounidenses (USD) con MOD, suministros (medicamentos/soluciones y materiales) necesarios en los procedimientos realizados por enfermeros superiores durante la fase de infusión de CMH en el TCMH autólogo criopreservado, según el coste directo medio (CDM), la desviación estándar (SD), el coste mínimo, el coste máximo, el primer cuartil, la mediana y el tercer cuartil y la mediana – São Paulo, SP, Brasil, 2025.

Procedimientos (número de observaciones)	CDM con MOD para enfermeros USD*	CDM con suministros (medicamentos/soluciones y materiales) USD*	Total CDM USD*	Desviación estándar USD*	Coste mínimo USD*	Coste máximo USD*	1.er cuartil USD*	Mediana USD*	Tercer cuartil USD*
1. Medición de constantes vitales, glucosa capilar y monitorización del paciente (n = 165)	0,49	0,03	0,52	0,27	0,19	1,86	0,38	0,38	0,56
2. Orientación y aclaración sobre el procedimiento de infusión de CMH (n = 19)	1,28	0,00	1,28	0,54	0,55	2,58	0,92	1,29	1,47
3. Orientación y asistencia con el control de la vejiga y los intestinos, y la ingesta de líquidos (n = 14)	0,82	1,32	2,14	0,62	1,22	3,38	1,59	1,96	2,54
4. Limpieza simultánea de muebles (n = 16)	1,06	0,78	1,85	0,39	1,13	2,42	1,49	1,69	1,90
5. Doble verificación y descongelación de bolsas de CMH (n = 90)	0,35	0,49	0,84	1,04	0,05	3,73	0,25	0,42	0,61
6. Infusión en bolsa CMH (n = 45)	3,27	628,36	631,62	1,66	628,10	637,13	630,68	631,23	632,23
7. Preparación y administración de medicamentos (IV) (n = 4)	2,67	0,90	3,57	1,83	0,88	4,88	2,06	3,40	4,48
8. Recopilación de pruebas de laboratorio (n = 4)	2,86	14,60	17,46	0,24	16,68	17,24	16,90	17,04	17,14
9. Lavado del equipo tras la infusión de CMH (n = 16)	1,91	0,84	2,76	0,68	1,01	3,77	1,51	1,84	2,34
10. Cambio de apósito de catéter venoso central, prueba de flujo y reflujo del catéter (n = 18)	0,97	0,45	1,41	0,35	0,37	2,11	1,21	1,36	1,41

*Tipo de cambio: 5,75 R$/1,00 USD. Banco Central, basado en el tipo de cambio medio de febrero a abril de 2025.

Entre los insumos consumidos en la fase de infusión de TCMH, la bolsa de CMH (627,91 dólares estadounidenses), el frasco anaeróbico (4,37 dólares estadounidenses) y la comadre (1,10 dólares estadounidenses) fueron los que tuvieron mayores costos unitarios.

La tercera fase, posterior a la infusión de TCMH, abarcó la observación sistemática de los procedimientos realizados tras el lavado del equipo, posterior a la infusión de CMH, contemplando todas las observaciones de los procedimientos ejecutados al final del turno vespertino, extendiéndose hasta la medianoche del día cero.

En la Tabla 3 se observa que, entre los siete procedimientos (100,00%) realizados, los mayores CDM totales fueron: Preparación y administración de medicamentos intravenosos y subcutáneos (6,77 dólares estadounidenses – DE = 2,06), Limpieza concurrente del mobiliario (2,40 dólares estadounidenses – DE = 0,35) y Asistencia para la eliminación vésico-intestinal, comidas y control hídrico (2,29 dólares estadounidenses – DE = 1,08). Teniendo en cuenta la suma de los CDM totales de los siete procedimientos, que dieron lugar a 463 observaciones, se obtuvo un CDM total de 16,84 dólares. En la mayoría de los procedimientos (85,71%), prevaleció el CDM con la MOD de enfermeros senior en la composición del CDM total.

**Tabla 3:** Distribución de los costes en dólares estadounidenses (USD) con MOD, insumos (medicamentos/soluciones y materiales) necesarios en los procedimientos realizados por enfermeros superiores en la fase posterior a la infusión del TCMH autólogo criopreservado, según el coste directo medio (CDM), la desviación estándar (SD), el coste mínimo, el coste máximo y la mediana – São Paulo, SP, Brasil, 2025.

Procedimientos (número de observaciones)	CDM con MOD para enfermeros USD*	CDM con suministros (medicamentos/soluciones y materiales) USD*	Total CDM USD*	Desviación estándar USD*	Coste mínimo en dólares estadounidenses*	Coste máximo en dólares estadounidenses*	1.er cuartil USD*	Mediana USD*	3.er cuartil USD*
1. Medición de signos vitales, glucosa capilar y peso corporal (n = 176)	0,77	0,03	0,80	0,26	0,38	2,27	0,58	0,74	0,93
2. Limpieza simultánea de muebles (n = 16)	1,11	1,30	2,40	0,35	1,65	2,81	1,99	2,20	2,55
3. Asistencia con la micción y la defecación, las comidas y la ingesta de líquidos (n = 77)	1,64	0,65	2,29	1,08	0,79	5,64	1,34	2,46	2,90
4. Vaciado y limpieza del equipo de calentamiento de líquidos (n = 16)	1,53	0,35	1,88	0,54	0,91	2,75	1,29	1,67	2,00
5. Preparación y administración de medicamentos intravenosos y subcutáneos (n = 55)	4,10	2,66	6,77	2,06	2,45	11,14	3,92	5,42	7,12
6. Preparación y administración de medicamentos orales (n = 110)	0,81	0,17	0,98	0,73	0,28	3,07	0,49	0,67	1,01
7. Cambio de apósito de catéter venoso central, prueba de flujo y reflujo del catéter (n = 13)	1,09	0,62	1,72	1,06	0,75	3,77	0,93	1,12	1,32

*Tipo de cambio: 5,75 R$/1,00 USD. Banco Central, basado en el tipo de cambio medio de febrero a abril de 2025.

En la fase posterior a la infusión de TCMH, los insumos de insulina regular (24,89 dólares), agua destilada 1000 ml (13,10 dólares) e insulina NPH (8,63 dólares) fueron los que tuvieron mayores costos unitarios. El equipo de macrogotas con inyector lateral y filtro de aire (2,81 dólares estadounidenses) fue el material más utilizado (42,19%) en la administración de las soluciones de hidratación.

## DISCUSIÓN

Todos los procedimientos realizados en el «día cero» del TCMH fueron ejecutados exclusivamente por enfermeros senior con competencia clínica especializada demostrada, lo que pone de manifiesto el compromiso del HPT de mantener un equipo altamente cualificado, con el fin de garantizar la seguridad de las etapas esenciales del TCMH. Cabe destacar la importancia de la actuación de enfermeros debidamente capacitados en este momento crítico del trasplante, ya que el éxito del procedimiento, la seguridad del paciente y la prevención o minimización de eventos adversos dependen directamente de la experiencia técnica de estos profesionales.

El enfermero desempeña un papel central en el cuidado del paciente sometido a TCMH, su experiencia técnica es determinante para la seguridad, la prevención de complicaciones y la realización cualificada de los procedimientos requeridos^(16,17)^.

En la fase previa a la infusión, el 59,07% del CDM total correspondió a MOD de enfermeros senior; los costos más significativos (25,55 dólares estadounidenses) se debieron a la desinfección y preparación del equipo calentador de líquidos. Cabe aclarar, en lo que respecta a las prácticas de infusión de CMH criopreservadas, que la descongelación de las bolsas requiere equipo hospitalario y técnicas cuidadosamente validadas (control riguroso de la temperatura, limpieza y desinfección adecuadas y técnica estéril), para garantizar la viabilidad celular, minimizar los riesgos de contaminación y garantizar la seguridad del receptor^(18)^.

La administración de medicamentos intravenosos y subcutáneos (CDM = 8,55 dólares estadounidenses) destacó en la composición del CDM total de la fase previa a la infusión, siendo un procedimiento necesario para el manejo clínico, a través de medidas farmacológicas destinadas a la prevención de eventos adversos (EA) relacionados con la infusión.

Un estudio que analizó 1269 infusiones de CMH autólogas criopreservadas, que contenían bolsas con el reactivo crioprotector dimetilsulfóxido (DMSO), en 1191 pacientes adultos, verificó que el 37,8% de las infusiones presentaron algún EA. El DMSO se asoció con la mayoría de las reacciones, lo que refuerza que las intervenciones clínicas, especialmente los ajustes y la administración de medicamentos antes de la infusión, pueden reducir la toxicidad y aumentar la seguridad del trasplante. La frecuencia de estos EA, clasificados como leves o moderados, indica la pertinencia de estrategias preventivas, como la hidratación adecuada y el uso de antihistamínicos y antieméticos, capaces de mitigar los riesgos y mejorar la seguridad de la infusión de CMH^(19)^. Sin embargo, entre los medicamentos utilizados en este estudio, la insulina regular (24,89 dólares) presentó el mayor CDM, debido a comorbilidades preexistentes que requirieron ajustes glicémicos para el manejo clínico seguro de los pacientes trasplantados.

Los CDM de la recolección de pruebas de laboratorio (5,50 dólares estadounidenses); la asistencia y la realización de la higiene corporal y la limpieza concurrente de la cama y el mobiliario (5,28 dólares estadounidenses) también repercutieron en el CDM total de la fase previa a la infusión. Una revisión sistemática^(20)^, que abarcó estudios realizados en unidades de trasplante de células hematopoyéticas, demostró que varios patógenos estaban directamente asociados con fuentes ambientales, brotes virales y prácticas inadecuadas de desinfección. Estos hallazgos indican que las buenas prácticas de higiene, limpieza y desinfección son una inversión esencial para evitar infecciones que pueden provocar un empeoramiento clínico, prolongar las hospitalizaciones y aumentar significativamente los costos de la atención^(20)^.

Entre los procedimientos realizados en la fase de infusión, la infusión de la bolsa de CMH (95,20%) presentó la mayor contribución a la composición del CDM total (663,45 USD – 100,00%), seguida de la recolección de pruebas de laboratorio (2,63%).

Un estudio^(21)^ que analizó el ciclo completo de recolección, criopreservación, almacenamiento y utilización de CMH en un centro de trasplantes europeo, a lo largo de 12 años, evidenció que la recolección, el procesamiento, el almacenamiento y el desecho de CMH implican costos sustanciales y a menudo subestimados, especialmente cuando se consideran las bolsas que nunca llegan a utilizarse en trasplantes.

Se observó que la infusión de CMH se produce principalmente en un breve intervalo después de la Recolección, y que el manejo de las bolsas almacenadas durante largos períodos de tiempo supone un elevado gasto para los servicios^(21)^.

Otro estudio^(22)^, que evaluó a 613 pacientes con mieloma múltiple durante nueve años, constató que la tasa de utilización de las bolsas almacenadas durante más de 30 días era extremadamente baja (14,9%) y que el 69,00% de los pacientes mantuvieron bolsas de CMH criopreservadas almacenadas durante dos años o más, con costos significativos asociados al mantenimiento en nitrógeno líquido, procedimientos adicionales de aféresis y eliminación tardía. Gran parte de los costos relacionados con el ciclo de las CMH no se concentran en el momento de la infusión, sino en las etapas previas de movilización, recolección y almacenamiento prolongado, procesos estandarizados que influyen directamente en el valor final de los insumos utilizados en el momento de la administración a pie de cama^(22)^.

Ambos estudios^(21,22)^ mencionados anteriormente destacan que las instituciones de salud necesitan revisar sus prácticas y flujos relacionados con la gestión de las CMH para optimizar los recursos, reducir el desperdicio y garantizar la sostenibilidad económica y operativa del TCMH.

En la fase de infusión de CMH, la aparición de reacciones adversas requirió la recolección de exámenes de laboratorio, y los frascos anaeróbicos para hemocultivo correspondieron a artículos de alto costo unitario. Un estudio de revisión narrativa destacó que la mayoría de los hemocultivos recolectados en el entorno hospitalario, con indicaciones basadas en fiebre o alteraciones inespecíficas, presentan una baja correlación con la contaminación bacteriana y enfatizó que la recolección indiscriminada contribuye al aumento de los costos^(23)^. Así, aunque la investigación de la fiebre durante la infusión de CMH es clínicamente pertinente, la indicación criteriosa del hemocultivo se vuelve esencial para equilibrar la precisión diagnóstica, la seguridad del paciente y la racionalidad en el uso de insumos, particularmente aquellos de alto costo unitario.

En la fase posterior a la infusión de CMH, la preparación y administración de medicamentos (6,77 USD – 40,20%) correspondió al mayor CDM. El material más utilizado en la administración de soluciones de hidratación fue el equipo de macrogotas con inyector lateral y filtro de aire (2,81 dólares estadounidenses), junto con el Suero fisiológico al 0,9% 1000 ml (1,07 dólares estadounidenses). Esto se debe a que una buena práctica adoptada en la UTCMH consiste en sustituir el equipo cada vez que se prepara una solución hidratante, junto con el uso de una bomba de infusión continua. Se justifica el énfasis en la hidratación después de la infusión de CMH criopreservadas en DMSO, ya que un estudio de revisión narrativa describió que la infusión de CMH con DMSO está relacionada con efectos nefrotóxicos, cardiovasculares, neurológicos y gastrointestinales^(24)^.

Un estudio^(25)^ investigó la variación de las prácticas de hidratación intravenosa tras la infusión de HME autólogas criopreservadas en DMSO en 510 centros de trasplantes vinculados al *European Bone Marrow Transplantation*. Los resultados demostraron que la hidratación posinfusión se utiliza ampliamente para proteger la función renal y ayudar a eliminar el DMSO del organismo; sin embargo, aún no hay evidencia científica que confirme su eficacia en la reducción de la toxicidad asociada^(25)^.

El segundo procedimiento con mayor CDM en la fase postinfusión fue la limpieza concurrente del mobiliario (2,40 dólares estadounidenses). Este valor es inferior al obtenido en la fase preinfusión de las HME (5,28 dólares estadounidenses), ya que en ella el costo no incluye el traslado y la asistencia para la higiene corporal del paciente.

A pesar de que el CDM con insumos fue el que más contribuyó a la composición del CDM total de la fase intra TCMH, en las tres fases prevaleció el CDM con la MOD de los enfermeros senior.

Estudios^(17,18,19,20,21,22,23,24,25,26)^ refuerzan que el enfermero es el profesional que actúa más cerca del paciente durante todas las fases del TCMH, que tiene una elevada complejidad y está impregnado de una intensa carga emocional y un alto riesgo clínico, desempeñando funciones que incluyen la vigilancia clínica, el manejo de los efectos adversos, el apoyo emocional y la educación terapéutica, que requieren una gran experiencia profesional^(17,18,19,20,21,22,23,24,25,26)^. Estas actividades, que también se observaron en este estudio, justifican por qué la MOD de los enfermeros representó la mayor parte del CDM.

Se evidencia que la atención de enfermería en TCMH tiene un impacto directo en la prevención de complicaciones, especialmente a través de la vigilancia clínica sistemática, la implementación de protocolos de seguridad y el monitoreo temprano de efectos toxicológicos e infecciosos, lo que demuestra que la estandarización de los procedimientos de enfermería en la infusión autóloga de células madre reduce la variabilidad asistencial y contribuye a resultados clínicos más seguros^(6)^. La presencia continua, la vigilancia sistemática, la ejecución de cuidados esenciales y la prevención de EA sitúan a los enfermeros en el núcleo operativo y asistencial de los cuidados requeridos en la TCMH^(6,16,26,27)^. Cabe destacar que los costos asociados a la actuación de los enfermeros no deben interpretarse como un indicador de onerosidad, sino como una inversión esencial para la seguridad del paciente y la calidad asistencial. De este modo, los hallazgos de este estudio corroboran la evidencia de que el tiempo dedicado por la enfermería a los cuidados directos es un componente indispensable para la seguridad del TCMH.

### Implicaciones Para la Práctica

Conocer los costos relacionados con la asistencia de enfermería en las diferentes fases/etapas del día cero del TCMH, especialmente en las instituciones públicas de salud en el contexto del Sistema Único de Salud, puede ayudar a formular estrategias que abarquen también los aspectos financieros, privilegiando la revisión de procesos y la asignación racional de recursos finitos, sin comprometer la calidad deseada, lo que se traduce en mejores resultados para los pacientes.

### Limitación del Estudio

Se señala como limitación la observación no participante restringida a los procedimientos relacionados con el TCMH autólogo en la modalidad criopreservada, lo que no permite la aplicación de los resultados obtenidos a otros tipos de TCMH o modalidades de infusión.

## CONCLUSIÓN

El CDM total de la asistencia prestada por enfermeros senior a pacientes en el día cero del TCMH, a partir de 1556 observaciones de 28 procedimientos, correspondió a 739,81 dólares estadounidenses (100,00%), siendo el 8,04% relativo a la fase pre-TCMH, el 89,68% a la fase intra-TCMH y el 2,28% a la fase post-TCMH. En las tres fases, predominó el CDM con la MOD de los enfermeros en la composición del CDM total. Sin embargo, en la fase intra TCMH, el CDM con insumos fue el que más contribuyó a la composición del CDM total, destacando la bolsa de CMH (valor unitario de 627,91 dólares).

## DISPONIBILIDAD DE DATOS

No aplicable. Se trata de un estudio de caso único cuyo conjunto de datos que respalda los resultados no está disponible públicamente debido a la presencia de información sensible y a la necesidad de preservar la confidencialidad de los participantes. Toda la información relevante para la comprensión de los hallazgos se describe en el cuerpo del artículo.
